# Toward a Common Terminology for the Gyri and Sulci of the Human Cerebral Cortex

**DOI:** 10.3389/fnana.2018.00093

**Published:** 2018-11-19

**Authors:** Hans J. ten Donkelaar, Nathalie Tzourio-Mazoyer, Jürgen K. Mai

**Affiliations:** ^1^Department of Neurology, Donders Center for Medical Neuroscience, Radboud University Medical Center, Nijmegen, Netherlands; ^2^IMN Institut des Maladies Neurodégénératives UMR 5293, Université de Bordeaux, Bordeaux, France; ^3^Institute for Anatomy, Heinrich Heine University, Düsseldorf, Germany

**Keywords:** terminology, gyri, sulci, cerebral cortex, human brain

## Abstract

The gyri and sulci of the human brain were defined by pioneers such as Louis-Pierre Gratiolet and Alexander Ecker, and extensified by, among others, Dejerine (1895) and von Economo and Koskinas (1925). Extensive discussions of the cerebral sulci and their variations were presented by Ono et al. (1990), Duvernoy (1992), Tamraz and Comair (2000), and Rhoton (2007). An anatomical parcellation of the spatially normalized single high resolution T1 volume provided by the Montreal Neurological Institute (MNI; Collins, 1994; Collins et al., 1998) was used for the macroscopical labeling of functional studies (Tzourio-Mazoyer et al., 2002; Rolls et al., 2015). In the standard atlas of the human brain by Mai et al. (2016), the terminology from Mai and Paxinos (2012) is used. It contains an extensively analyzed individual brain hemisphere in the MNI-space. A recent revision of the terminology on the central nervous system in the *Terminologia Anatomica* (TA, 1998) was made by the Working Group Neuroanatomy of the Federative International Programme for Anatomical Terminology (FIPAT) of the International Federation of Associations of Anatomists (IFAA), and posted online as the *Terminologia Neuroanatomica* (TNA, 2017: http://FIPAT.library.dal.ca) as the official FIPAT terminology. This review deals with the various terminologies for the cerebral gyri and sulci, aiming for a common terminology.

## Introduction

Although the gyri and sulci of the human brain were already beautifully illustrated by Vicq d'Azyr (1786) and von Soemmerring (1791), they were named and defined by Gratiolet (1854), Huschke (1854), Ecker (1869), Pansch (1868, 1879), Jensen (1871), Wernicke (1876), Eberstaller (1884, 1890), and Brissaud (1893), and extensified by, among others, Dejerine (1895), Retzius (1896), von Economo and Koskinas (1925), and Rose (1935). More recently, extensive discussions of the cerebral sulci and their variations were presented by Ono et al. (1990), Duvernoy (1992), Tamraz and Comair (2000), and Rhoton (2007). An anatomical parcellation of the spatially normalized single high resolution T1 volume provided by the Montreal Neurological Institute (MNI) was used for the macroscopical labeling of functional studies (Tzourio-Mazoyer et al., 2002; Rolls et al., 2015), using largely the Dejerine terminology. The previously much used Talairach atlas (Talairach and Tournoux, 1988) proved to be rather inaccurate for the cytoarchitectonic allocation of functional activations (Tzourio-Mazoyer et al., 2002; Eickhoff et al., 2005). In the standard atlas of the human brain by Mai et al. (2016), the terminology from Mai and Paxinos (2012) is used. It contains an individual brain hemisphere in the MNI-space. In a recent pocket atlas (Mai and Majtanik, 2017), a probabilistic neuroanatomy of 152 individuals was presented to which the main atlas is registered. Mai and colleagues used the Brodmann (1909) and von Economo and Koskinas (1925) subdivisions of the cerebral cortex. A comprehensive cellular-resolution atlas of the adult human brain (Ding et al., 2016) presents the first digital human brain atlas across a complete adult female brain. The terminology used largely follows Brodmann terminology.

Recently, a revision of the terminology on the central nervous system in the *Terminologia Anatomica* (TA, 1998) was made by the Working Group Neuroanatomy of the Federative International Programme for Anatomical Terminology (FIPAT) of the International Federation of Associations of Anatomists (IFAA), and posted online as the *Terminologia Neuroanatomica* (TNA, 2017: http://FIPAT.library.dal.ca; for an introductory paper, see ten Donkelaar et al., 2017) as the official FIPAT terminology. This review deals with the various terminologies for the cerebral gyri and sulci on the superolateral, inferomedial, and basal surfaces of the cerebrum, aiming for a common terminology. It combines the data from the TNA (2017), an illustrated version (ten Donkelaar et al., 2018) and additional terms found in preparing this review.

## Brief review of the literature

**Figure 1:**
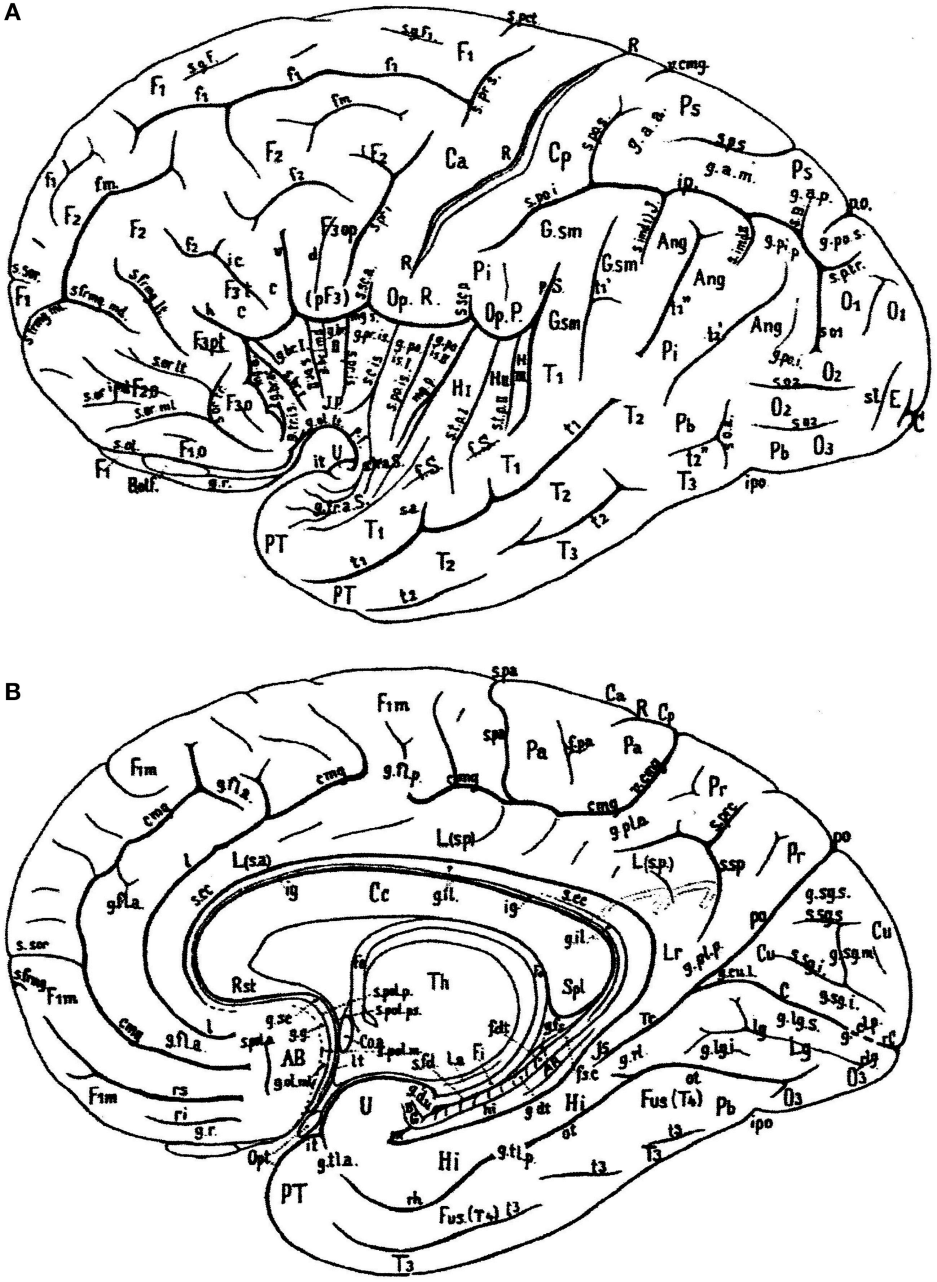
Sulcal pattern in the human cerebral cortex: **(A)** Lateral aspect; **(B)** medial aspect (after von Economo and Koskinas, 1925). *AB*, area parolfactoria of Broca; *Ang*, angular lobule; *AR*, gyri of Andreas Retzius; *BB*, band of Broca; *BG*, bandelette of Giacomini; *B.olf*, olfactory bulb; *C*, calcarine fissure; *Ca, Cp*, anterior and posterior central gyri; *Cc*, corpus callosum; *Coa*, anterior commissure; *Cu*, cuneus; *cmg*, callosomarginal sulcus; *d*, diagonal sulcus of Eberstaller; *E*, descending occipital gyrus of Ecker; *F1, F2, F3*, first, second and third frontal gyri; *F3o, F3op, F3pt, F3t*, orbital, opercular, pretriangular, and triangular parts of F3; *f1, f2*, superior and inferior frontal sulci; *f.dt*, fascia dentata; *f.m*, middle frontal sulcus; *fo*, fornix; *f.pa*, paracentral fossa; *fs.c*, fasciola cinerea; *f.Sy*, Sylvian fissure; *Fus (T4)*, fusiform gyrus; *g.ant.a, g.ant.d, g.ant.prc*, anticentral, antidiagonal and antiprecentral gyrus of operculum; *Gsm*, supramarginal lobule; *g.a.a., g.a.m., g.a.p.*, arcuate gyri of anterior, middle and posterior superior parietal lobule; *g.amb*, gyrus ambiens; *g.br.a., g.br.I, II, III, g.br.imd*, accessory short, first, second and third short and intermediate short gyri of insula; *g.cl.p.*, posterior cuneolingual gyrus; *g.dt*, dentate gyrus; *g.d.u.*, digital gyri of uncus; *g.fl.a., g.fl.p.*, anterior and posterior frontolimbic gyri; *g.fs*, fasciolar gyrus; *g.g*, geniculate gyrus; *g.il*, intralimbic gyrus; *g.lg.i, g.lg.s*, inferior and superior lingual gyri; *g.ol.lt, g.ol.ml*, lateral and medial olfactory gyri; *g.pip*, posterior inferior parietal gyrus; *g.pl.a, g.pl.p*, anterior and posterior parietolimbic gyrus; *g.po.i, g.po.s*, inferior and superior parieto-occipital gyrus; *g.po.is.I, g.po.is.II, first* and second postcentral gyrus of insula; *g.pr.is*, precentral gyrus of isthmus; *g.r*, straight gyrus; *g.rl*, retrolimbic gyrus; *g.sc*, subcallosal gyrus; *g.sg.i, g.sg.m, g.sg.s*, inferior, middle, and superior sagittal gyrus of cuneus; *g.sml*, semilunar gyrus; *g.str*, subtriangular gyrus of operculum; *g.tl.a, g.tl.p*, anterior and posterior temporolimbic gyrus; *g.tr.a.S*, anterior transverse temporal gyri of Schwalbe; *g.tr.is*, transverse gyrus of insula; *g.tr.op.I, g.tr.op.II, g.tr.op.III*, first, second and third transverse gyrus of parietal operculum; *H.I, H.II, first* and second gyrus of Heschl; *Hi*, hippocampal gyrus; *h*, horizontal branch of Sylvian fissure; *hi*, hippocampal fissure; *Is*, isthmus; *ic*, incisura capiti; *ig*, indusium griseum; *ip*, interparietal sulcus; *ipo*, preoccipital incisure; *it*, temporal incisure; *Lg*, lingula; *L.s.a, L.s.p*, anterior and posterior part of superior limbic gyrus; *Lr*, retrosplenial part of limbic gyri; *l*, intralimbic sulcus; *l.a*, lamina affixa; *l.g*, lingual sulcus; *lt*, lamina terminalis; *mg.a, mg.p*, anterior and posterior margin of circular sulcus of insula; *O1, O2, O3*, first, second and third occipital gyrus; *Op.P*, parietal operculum; *Op.R*, frontal operculum of Rolando; *Opt*, optic nerve; *ot*, occipitotemporal (collateral) fissure; *Pa*, paracentral lobule; *Pb*, basal parietal region; *Pi*, inferior parietal lobule; *Pr*, precuneus; *Ps*, superior parietal lobule; *PT*, temporopolar gyrus; *p.f*, falciform incisure; *po*, parieto-occipital fissure; *p.Sy*, posterior branch of Sylvian fissure; *R*, sulcus of Rolando; *Rst*, rostrum of corpus callosum; *rC*, retrocalcarine fissure; *rh*, rhinal fissure; *ri, rs*, inferior and superior rostral sulcus; *rl*, retrolingual sulcus; *Spl*, splenium of corpus callosum; *s.a*, acoustic sulcus; *s.B*, sulcus of Brissaud; *s.br.I, s.br.II, first* and second short sulcus of insula; *s.cc*, sulcus of corpus callosum; *s.c.is*, central sulcus of insula; *s.fd*, fimbriodentate sulcus; *s.frmg.ml, s.frmg.md, s.frmg.lt*, medial, middle, and lateral frontomarginal sulcus; *s.g.F1*, sulcus of first frontal gyrus; *s.imdI, s.imdII, first* (of Jensen) and second (of Eberstaller) intermediate sulcus; *s.l*, lunate sulcus; *so1, so2, first* and second occipital sulcus; *s.ol* olfactory sulcus; *s.or.imd, s.or.lt, s.or.ml, s.or.tr*, intermediate, lateral, medial, and transverse orbital sulcus; *s.pa*, paracentral sulcus; *s.po.i, s.po.s*, inferior and posterior postcentral sulcus; *s.po.is* postcentral sulcus of isthmus; *s.pol.a, s.pol.m, s.pol.p, s.pol.ps*, anterior, middle, posterior, and postremal paraolfactory sulcus; *s.prc*, precuneate sulcus; *s.prd*, prediagonal sulcus; *s.pr.i, s.pr.s*, inferior and superior precentral sulcus; *s.pr.is*, precentral sulcus of insula; *s.p.s, s.p.tr*, superior and transverse parietal sulcus; *s.rh.i*, internal rhinal sulcus; *s.san*, semianular sulcus; *s.sc.a, s.sc.p*, anterior and posterior subcentral sulcus; *s.sg.i, s.gs.s*, inferior and superior sagittal sulcus of cuneus; *s.so*, suboccipital sulcus; *s.sor*, supraorbital sulcus; *s.sp*, subparietal sulcus; *s.tp.I, s.tp.II, first* and second deep temporal sulcus; *s.tr.a.S*, anterior transverse temporal sulci of Schwalbe; *s.tr.op.I, s.tr.op.II, first* and second transverse sulcus of parietal operculum; *T1, T2, T3*, first, second and third temporal sulcus; *Th*, thalamus; *Tr*, trunk of the parieto-occipital and calcarine fissures; *Tr.o*, olfactory trigonum; *Tu.o*, olfactory tubercle; *t1, t2, t3*, first, second and third temporal sulci; *U*, uncus; *v*, ventral branch of the Sylvian fissure; *v.cmg*, vertical branch of callosomarginal sulcus.

In Figure 1, the wealth of gyri and sulci of the human cerebral cortex as distinguished by von Economo and Koskinas (1925) is shown. The gyri of the cerebral lobes are indicated by the classical numbering such as F1-F3, T1-T4, and the sulci without capitals (f1, f2, etc). Clearly visible are the first and second intermediate parietal sulci of Jensen and Eberstaller (s.imdI and s.imdII, respectively) as well as the frontomarginal sulcus of Wernicke with various components. Many of the smaller or infrequent sulci were forgotten, several of which were reintroduced in the recent human brain mapping era and in the TNA. The Supplementary Table 1 contains a list of synonyms and eponyms for the cerebral gyri and the Supplementary Table 2 those of the main sulci.

**Figure 2:**
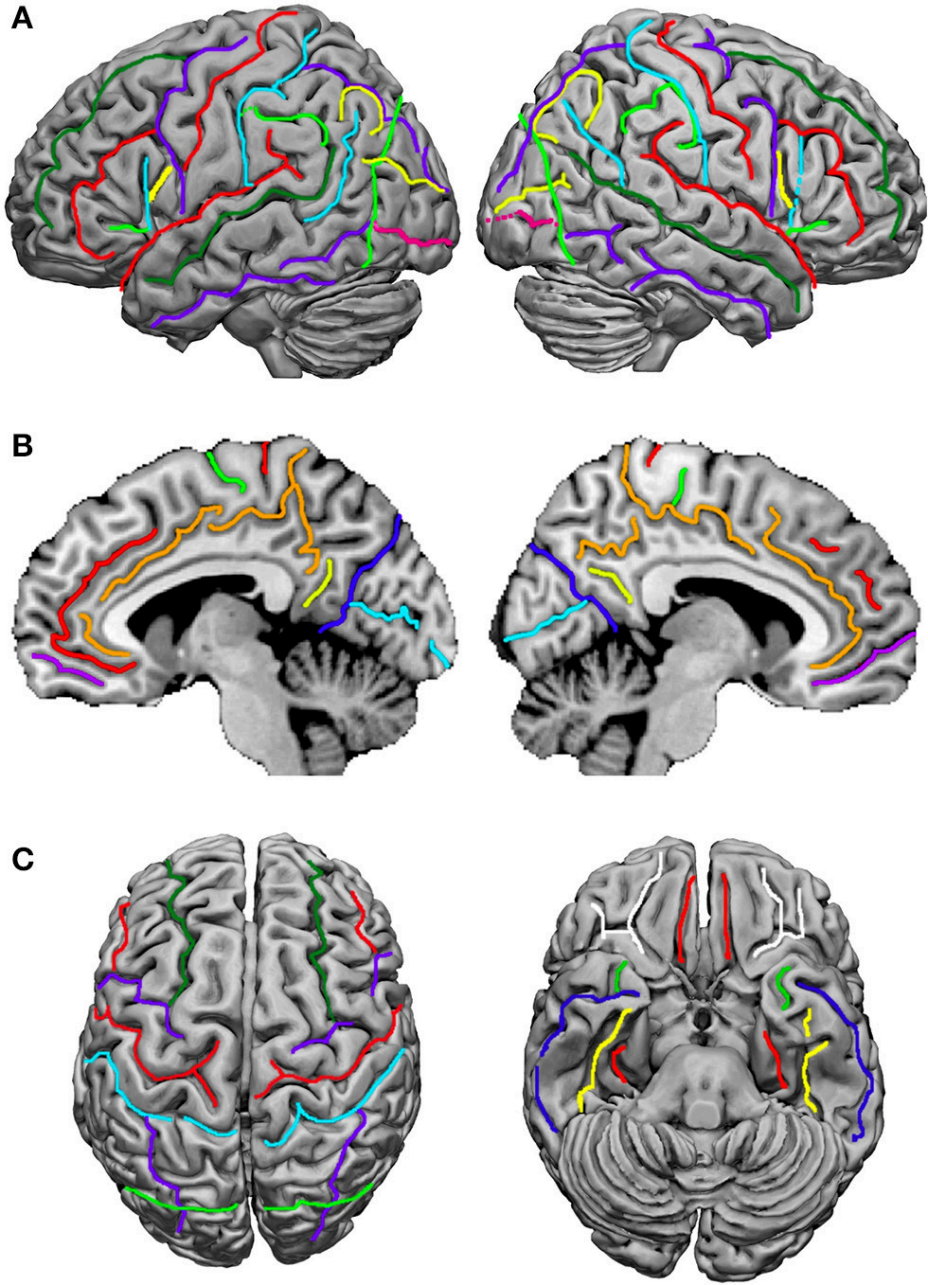
Sulci definition on the 3-D renderings of the T1 MNI single subject brain: **(A)** Lateral view; left hemisphere on the left. From the frontal pole to the occipital pole, the following sulci are indicated: the superior frontal sulcus (*dark green*), the inferior frontal sulcus (*red*), the anterior, horizontal ramus of the lateral, Sylvian sulcus (*light green*), the ascending, vertical ramus of the lateral sulcus (*cyan*), the diagonal sulcus (*yellow*), the precentral sulcus (*purple*), the central, Rolandic sulcus (*red*), the postcentral sulcus (*cyan*), the intraparietal and intraoccipital sulci (*purple*), the anterior limit of the occipital lobe, corresponding in its inferior part to the anterior occipital sulcus (*light green*), the transverse occipital sulcus (*yellow*), and the inferior occipital sulcus (*pink*). **(B)** medial view; from the frontal to the occipital pole: the paracingulate sulcus (*red*), the cingulate sulcus (*orange*), the anterior rostral sulcus (*purple*), the paracentral sulcus (*light green*), the central, Rolandic sulcus (*red*), the marginal ramus (*orange*), the subparietal sulcus (*yellow*), the parieto-occipital sulcus (*blue*), and the calcarine sulcus (*cyan*). **(C)** At the left, superior view: the superior frontal sulcus (*dark green*) runs in the same direction and is symmetric in an horizontal plane with the intraoccipital sulcus (*purple*); the central, Rolandic sulcus (*red*), the precentral sulcus (*purple*), and the postcentral sulcus (*cyan*) run parallel. At the right, basal view: in the frontal lobe the orbital (*white*) and olfactory (*red*) sulci are depicted, and in the temporal lobe, the rhinal sulcus (*light green*), the inferior temporal sulcus (*dark blue*), the occipitotemporal sulcus (*yellow*) and the collateral sulcus (*red*). Adapted from Tzourio-Mazoyer et al. (2002).

Terminological differences used in Tzourio-Mazoyer's approach (Tzourio-Mazoyer et al., 2002; Rolls et al., 2015; Figure 2) vs. the *Terminologia Anatomica* (TA, 1998) concern the use of eponyms such as Rolandic operculum, Sylvian fissure and Heschl's gyrus, and the use of gyrus instead of lobule for the superior and inferior parietal lobules.

In the atlas of Mai et al. (2016) and the recent pocket atlas by Mai and Majtanik (2017), the use of the term fissure is advocated for the lateral, parietooccipital and hippocampal sulci. In the BNA (1895), the terms fissurae cerebri lateralis, collateralis, parietooccipitalis, calcarina, and hippocampi were used. In the JNA (1936), only the lateral, Sylvian fissure remained as fissure. This was corrected in the PNA (1955) and later editions, and for the cerebrum, the term fissure is in use only for the interhemispheric fissure. Therefore, the term fissure should not have been advocated anymore.

**Figure 3:**
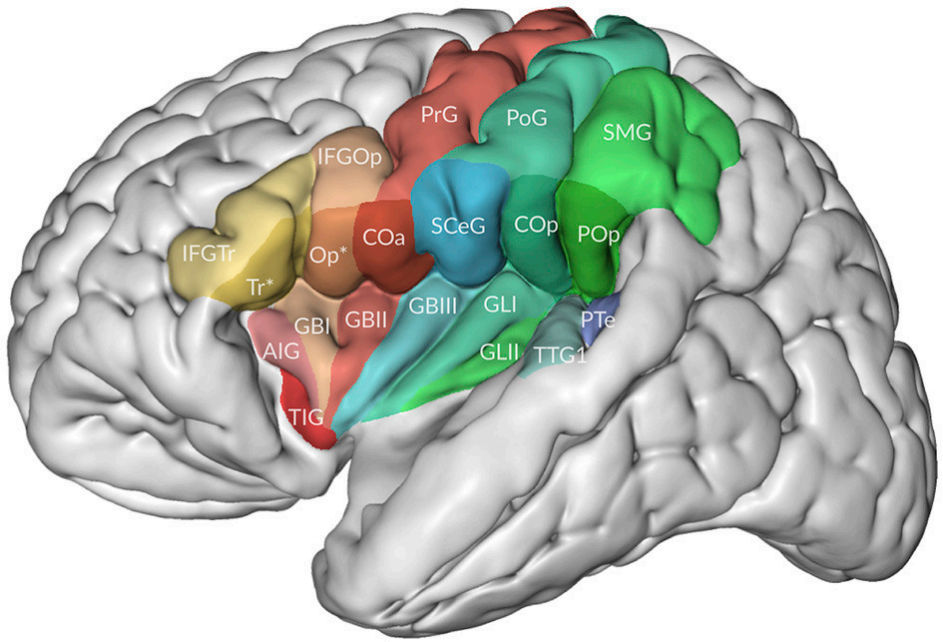
The relation of the opercula to the insula. Adapted from Mai and Majtanek (2017; kindly provided by Milan Majtanik, Düsseldorf). *AIG*, accessory insular gyrus; *COa, Cop*, anterior (precentral) and posterior (postcentral) opercula; *GBI-III*, short insular gyri; *GLI-II*, long insular gyri; *IFGOp*, with Op*, *IFGTr*, with Tr* opercular and triangular parts of inferior frontal gyrus with opercular and triangular parts of frontal operculum, *PoG*, postcentral gyrus; *POp*, parietal operculum; *PrG*, postcentral gyrus; *PTe*, planum temporale; *SCeG*, subcentral gyrus; *SMG*, supramarginal gyrus; *TIG*, transverse insular gyrus; *TTG1*, anterior transverse temporal gyrus.

Minor differences in Mai et al. (2016) are the use of the terms central operculum for the subcentral gyrus, anterior intermediate parietal sulcus for the first intermediate parietal sulcus of Jensen (see also Zlatkina and Petrides, 2014), medial occipitotemporal gyrus as a common term for the lingual gyrus and the parahippocampal gyrus, periinsular sulcus for the circular sulcus of the insula, and a rather extensive terminology for the opercula, including frontal, frontoparietal, and temporal opercula (Figure 3). Their frontoparietal operculum includes the anterior central (precentral) operculum, the subcentral gyrus, the posterior central (postcentral) operculum, and the parietal operculum. The first three collectively may belong to the subcentral gyrus.

**Figure 4:**
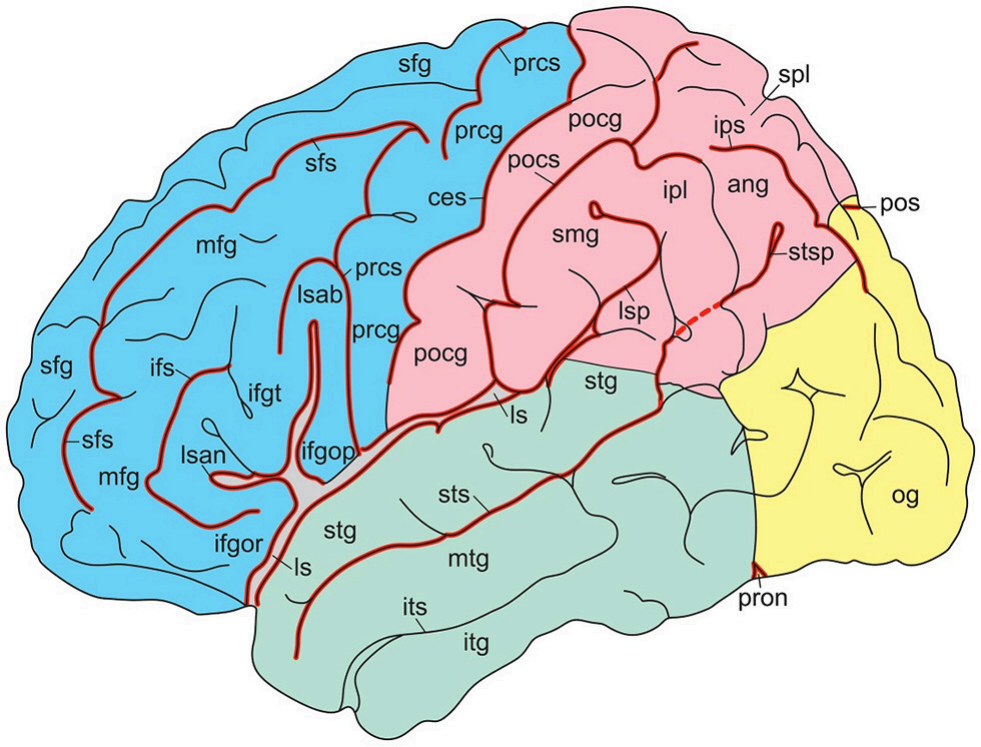
Gyri and sulci of the lateral aspect of the standard brain (from Nieuwenhuys et al., 2015; with permission from Springer Nature). *ang*, angular gyrus; *ces*, central sulcus; *ifgop, ifgor, ifgt*, inferior frontal gyrus, orbital, opercular, and triangular parts; *ifs*, inferior frontal sulcus; *ipl*, inferior parietal lobule; *ips*, intraparietal sulcus; *itg*, inferior temporal gyrus; *its*, inferior temporal sulcus; *ls, lsab, lsan, lsp*, lateral sulcus with ascending, anterior, and posterior branches; *mfg*, middle temporal gyrus; *mtg*, middle temporal sulcus; *og*, occipital gyri; *pocg*, postcentral gyrus; *pocs*, postcentral sulcus; *pos*, parietooccipital sulcus; *prcg*, precentral gyrus; *prcs*, precentral sulcus; *pron*, preoccipital notch; *sfg*, superior frontal gyrus; *sfs*, superior frontal sulcus; *smg*, supramarginal gyrus; *spl*, superior parietal lobule; *stg*, superior temporal gyrus; *sts, stsp*, superior temporal sulcus with posterior part.

**Figure 5:**
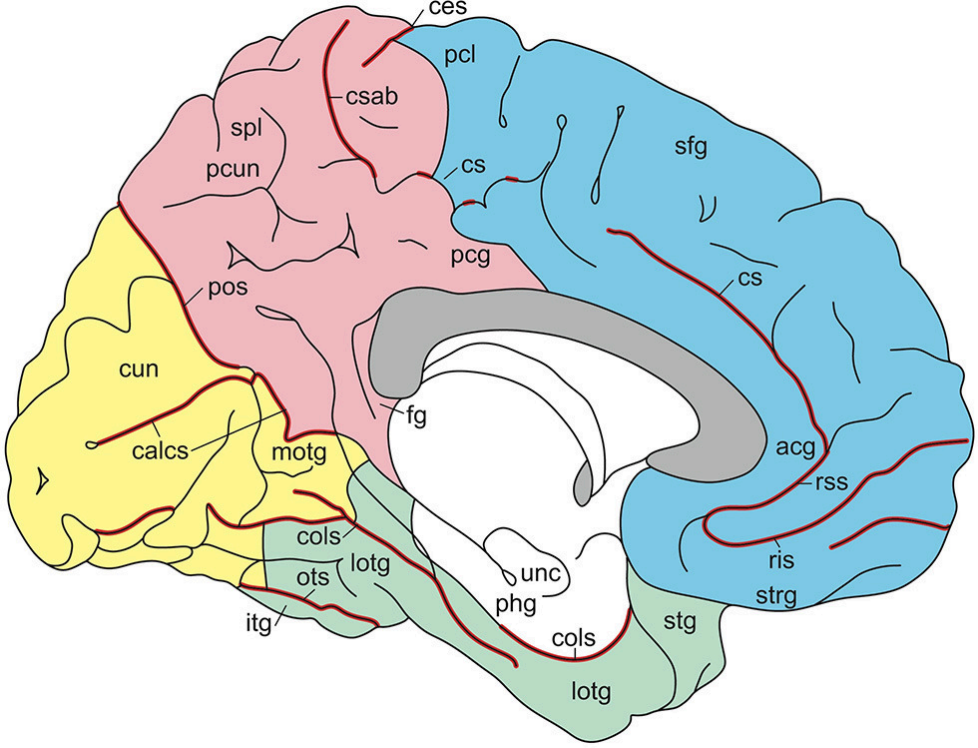
Gyri and sulci of the medial aspect of the standard brain (from Nieuwenhuys et al., 2015; with permission from Springer Nature). *acg*, anterior cingulate gyrus; *calcs*, calcarine sulcus; *ces*, central sulcus; *cols*, collateral sulcus; *cs, csab*, cingulate sulcus with ascending branch; *cun* cuneus; *fg*, fasciolar gyrus; *itg*, inferior temporal gyrus; *lotg*, lateral occipitotemporal gyrus (fusiform gyrus); *motg*, medial occipitotemporal gyrus (lingual gyrus); *ots*, occipitotemporal sulcus; *pcg*, posterior cingulate gyrus; *pcl*, paracentral lobule; *pcun*, precuneus; *phg*, parahippocampal gyrus; *pos*, parietooccipital sulcus; *ris, rss*, rostral inferior and rostral superior sulcus; *sfg*, superior frontal gyrus; *spl*, superior parietal lobule; *stg*, superior temporal gyrus; *strg*, straight gyrus; *unc*, uncus.

In their atlas of the human brain in MNI space, Mai et al. (2016) presented photographs of cell-stained sections of the right hemisphere of a 24-year-old male from the Vogt-collection in Düsseldorf (Vogt and Vogt, 1919). Schematic drawings show delineations of the cortex, which are based on the original maps of Brodmann (1909). The surface-based maps by Van Essen (2005); Van Essen et al. (2012) were modified by manually estimating areal boundaries on the atlas drawing and transforming them on the surface of the 3D reconstruction. Nieuwenhuys et al. (2015) adapted the standard brain, generated from the colin27 brain (http://www.bic.mni.mcgill.ca/ServicesAtlases/Colin27). In Figures 4, 5, gyri and sulci are shown for the lateral and medial aspects, respectively. The colin27 image is the result of averaging 27 linearly registered high-resolution T1-weighted scans of the same individual (Collins, 1994; Collins et al., 1998; Holmes et al., 1998), matched to the MNI305-space (Mazziota et al., 2001). Several neuroimaging software systems adopted the colin27 template as the standard reference. Nieuwenhuys et al. (2015) noted a few peculiarities of the colin27 template brain: (1) the Broca area of the inferior frontal gyrus is very large, but the middle frontal gyrus is relatively narrow; (2) the superior temporal sulcus is not continuous with the groove marking the cortex of the angular gyrus; (3) both the collateral and cingulate sulci are interrupted, and the posterior part of the cingulate sulcus shows an unusual zigzag course; and (4) the upper surface of the splenium of the corpus callosum has a remarkable bump. It may be added that no attempt was made to subdivide the lateral aspect of the occipital lobe, and that the fairly constant frontomarginal sulcus is absent.

In this review, the terminology of the recent TNA (2017) is presented along with short descriptions and currently used synonyms, and summarized in Tables 1–3. Both English and Latin official terms from the TNA are used. The sulci of the cerebral cortex can be divided into **interlobar sulci**, separating the cerebral lobes, and **lobar sulci** present in a lobe.

**Table 1:** Sulci and on the superolateral surface of the cerebral hemisphere (based on TNA, 2017; ten Donkelaar et al., 2018).

**English official terms and synonyms**	**Latin official terms and synonyms**	**Abbreviations and acronyms**	**Eponyms**
**Superolateral interlobar sulci**	**Sulci interlobares superolaterales**	
central sulcus	sulcus centralis	ces	sulcus of Rolando
lateral sulcus	sulcus lateralis	ls	sulcus of Sylvius
posterior ramus	ramus posterior	lsp
ascending ramus	ramus ascendens	lsas
anterior ramus	ramus anterior	lsan
parietooccipital sulcus	sulcus parietooccipitalis	pos	sulcus of Gratiolet
preoccipital notch	incisura preoccipitalis	pn	incisure of Meynert
**Frontal lobe**	**Lobus frontalis**	
frontomarginal sulcus	sulcus frontomarginalis	fmgs	sulcus of Wernicke
frontal pole	polus frontalis	FP
frontopolar area	area frontopolaris	FPA
superior frontopolar gyrus	gyrus frontopolaris superior	SFPG
middle frontopolar gyrus	gyrus frontopolaris medius	MFPG
inferior frontopolar gurus	gyrus frontopolaris inferior	IFPG
frontomarginal gyrus	gyrus frontomarginalis	FMG
frontal operculum	operculum frontale	FOp
inferior frontal gyrus	gyrus frontalis inferior	IFG; F3
orbital part	pars orbitalis	IFGOr
triangular part	pars triangularis	IFGTr	area of Broca
radiate sulcus	sulcus radiatus	ras	sulcus of Eberstaller
opercular part	pars opercularis	IFGOp	area of Broca
diagonal sulcus	sulcus diagonalis	dis	sulcus of Eberstaller
inferior frontal sulcus	sulcus frontalis inferior	ifs; f2
middle frontal gyrus	gyrus frontalis medius	MFG; F2
precentral gyrus	gyrus precentralis	PRG
precentral sulcus	sulcus precentralis	prs
anterior subcentral sulcus	sulcus subcentralis anterior	ascs
subcentral gyrus	gyrus subcentralis	SCeG	central or Rolandic operculum
posterior subcentral sulcus	sulcus subcentralis posterior	pscs
superolateral superior frontal gyrus	gyrus frontalis superior superolateralis	SFGL; F1
superior frontal sulcus	sulcus frontalis superior	sfs; f1
**Parietal lobe**	**Lobus parietalis**	
postcentral gyrus	gyrus postcentralis	POG
postcentral sulcus	sulcus postcentralis	pcs
superior parietal lobule	lobulus parietalis superior	SPL; P1
parietooccipital arc	arcus parietooccipitalis	POcA	first parietooccipital passage of Gratiolet
intraparietal sulcus	sulcus intraparietalis	ips
first intermediate sulcus; anterior intermediate sulcus	sulcus intermedius primus; sulcus intermedius anterior	fis	sulcus of Jensen
second intermediate sulcus; posterior intermediate sulcus	sulcus intermedius secundus; sulcus intermedius posterior	sis	sulcus of Eberstaller
transverse parietal sulcus	sulcus parietalis transversus	tps	sulcus of Brissaud
inferior parietal lobule	lobulus parietalis inferior	IPL; P2
angular gyrus	gyrus angularis	AG
parietal operculum	operculum parietale	POp
supramarginal gyrus	gyrus supramarginalis	SMG
**Occipital lobe**	**Lobus occipitalis**	
occipital pole	polus occipitalis	OP
lunate sulcus	sulcus lunatus	lus
transverse occipital sulcus	sulcus occipitalis transversus	tos
superior occipital gyrus	gyrus occipitalis superior	SOG; O1
middle occipital gyrus	gyrus occipitalis medius	MOG; O2
inferior occipital gyrus	gyrus occipitalis inferior	IOG; O3
descending occipital gyrus	gyrus occipitalis descendens	DOG	gyrus of Ecker
**Temporal lobe**	**Lobus temporalis**	
temporal pole	polus temporalis	TP
temporopolar cortex	cortex temporopolaris	TPC
superior temporal gyrus	gyrus temporalis superior	STG; T1
anterior part	pars anterior	STGa
posterior part	pars posterior	STGp	area of Wernicke
temporal operculum	operculum temporale	TOp
polar plane	planum polare	PPo
transverse temporal gyri	gyri temporales transversi		gyri of Heschl
anterior transverse temporal gyrus	gyrus temporalis transversus anterior	TTGa
posterior transverse temporal gyrus	gyrus temporalis transversus posterior	TTGp
temporal plane	planum temporale	PTe
transverse temporal sulci	sulci temporales transversi	
anterior transverse temporal sulcus	sulcus temporalis transversus anterior	atts
intermediate transverse temporal sulcus	sulcus temporalis transversus intermedius	itts
posterior transverse temporal sulcus	sulcus temporalis transversus posterior	ptts
superior temporal sulcus	sulcus temporalis superior	sts; t1
middle temporal gyrus	gyrus temporalis medius	MTG; T2
inferior temporal sulcus	sulcus temporalis inferior	its; t2
superolateral inferior temporal gyrus	gyrus temporalis inferior superolateralis	ITGL; T3
**Insula; insular lobe**	**Insula; lobus insularis**	Ins
insular gyri	gyri insulae	
long gyrus of insula	gyrus longus insulae	LGI
short gyri of insula	gyri breves insulae	SGI
transverse insular gyrus	gyrus transversus insulae	TIG
central sulcus of insula	sulcus centralis insulae	csi
circular sulcus of insula; periinsular sulcus	sulcus circularis insulae	cas	sulcus of Reil
limen insulae; insular threshold; frontotemporal junction	limen insulae; junctio frontotemporalis	LI

**Figure 6:**
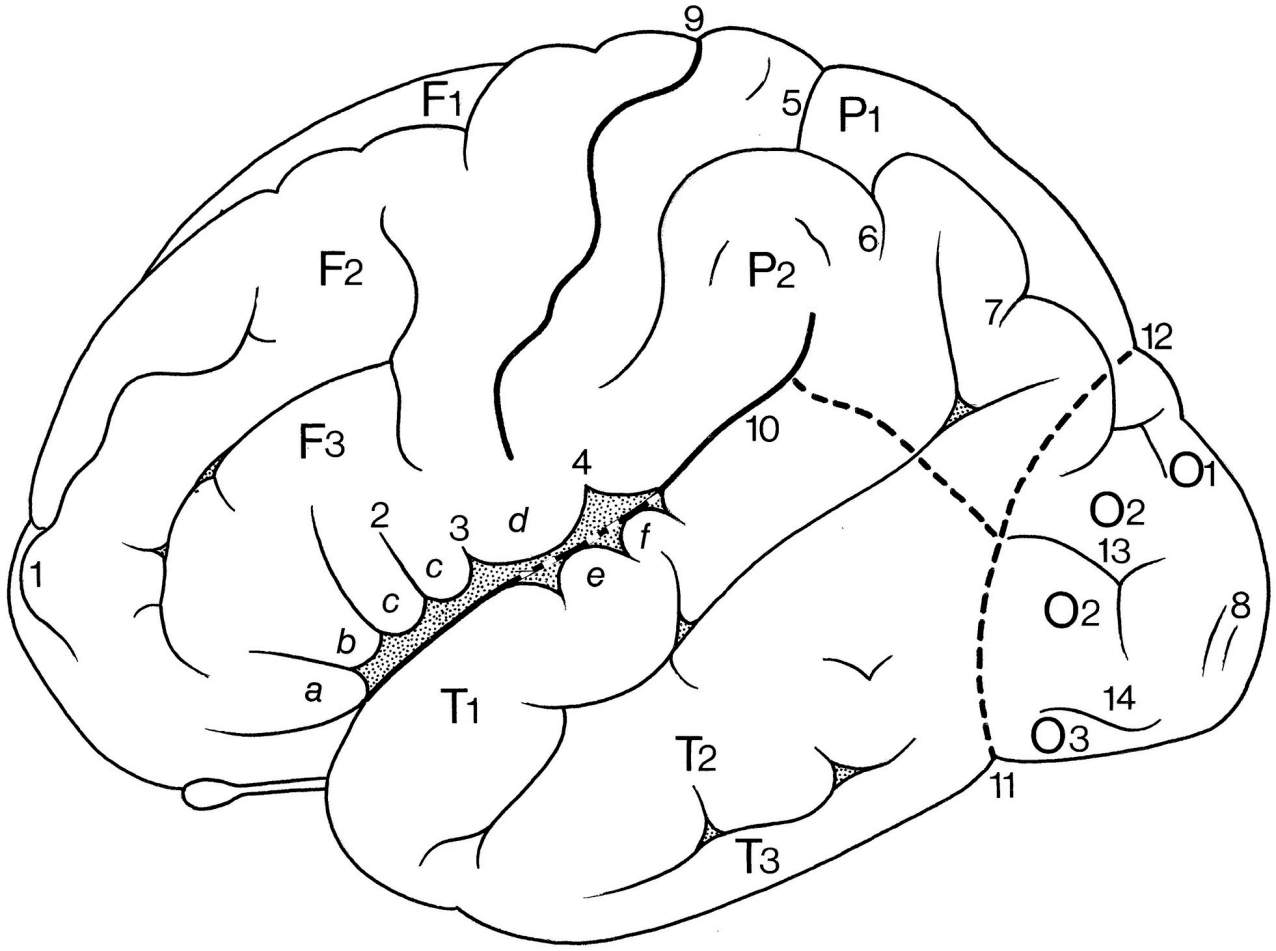
Gyri and sulci on the superolateral surface of the cerebral hemisphere (after Duvernoy, 1992; ten Donkelaar et al., 2018). *a, b, c*, orbital, triangular, and opercular (divided into two parts by *2*) parts of the inferior frontal gyrus; *d*, subcentral gyrus; *e, f*, transverse temporal gyri of Heschl; *F1, F2, F3*, superior, middle, and inferior frontal gyri; *O1, O2, O3*, superior, middle, and inferior occipital gyri; *P1, P2*, superior and inferior parietal lobules; *T1, T2, T3*, superior, middle, and inferior temporal gyri; *1*, frontomarginal sulcus of Wernicke; *2*, diagonal sulcus of Eberstaller; *3*, anterior subcentral sulcus; *4*, posterior subcentral sulcus; *5*, transverse parietal sulcus of Brissaud; *6*, first intermediate sulcus of Jensen; *7*, second intermediate sulcus of Eberstaller; *8*, descending occipital gyrus of Ecker; *9*, central sulcus; *10*, lateral sulcus; *11*, preoccipital notch of Meynert; *12*, parietooccipital sulcus; *13*, lunate sulcus; *14*, inferior occipital sulcus.

*For a summarizing figure, see Figure 6*.

## Superolateral surface of the cerebral hemisphere

The lateral aspect of the cerebrum (Figure 6; and Table 1) shows two **interlobar sulci**: the lateral and central sulci. The *lateral sulcus* (*sulcus lateralis* of Sylvius), known for a long time as the *Sylvian fissure*, between the frontal and temporal lobes, has three branches: the *anterior* (*ramus anterior*) or *horizontal ramus*, the *ascending* (*ramus ascendens*) or *vertical ramus* and the *posterior ramus* (*ramus posterior*), separating the parietal and temporal lobes. The *central sulcus* (*sulcus centralis* of Rolando) separates the frontal and parietal lobes. It is not a straight line but forms two arches from the superior margin of the hemisphere downwards to the lateral sulcus, the genu superior and the genu inferior (Broca, 1878a). The upper arch borders a “knob,” which protrudes posteriorly, and contains the hand area of the somatosensory cortex (Rumeau et al., 1994; Yousry et al., 1997). The *parietooccipital sulcus* (*sulcus parietooccipitalis* of Gratiolet) indicates the border between the parietal and occipital lobes superiorly, and the *preoccipital notch* (*incisura preoccipitalis* of Meynert) marks that border inferiorly.

The **frontal lobe** (**lobus frontalis**) shows the following **gyri**: the *superior, middle* and *inferior frontal gyri* (*gyrus frontalis superior*, -*medius*, and -*inferior*), classically numbered F1, F2, and F3, separated by *superior* and *inferior frontal sulci* (*sulcus frontalis superior* and-*inferior*, classically numbered f1 and f2, see Figure 1), and the *precentral gyrus* (*gyrus precentralis*). The central sulcus usually does not reach the lateral sulcus, and is separated from it by a short gyrus, the *subcentral gyrus* (*gyrus subcentralis*), delimited in front and behind by the *anterior* and *posterior subcentral sulci* (*sulcus subcentralis anterior* and-*posterior*), respectively, as distinguished by Dejerine (1895); Testut and Latarjet (1948). The subcentral gyrus is also known as the central or Rolandic operculum. The inferior frontal gyrus comprises three parts, *orbital, triangular* and *opercular* (*pars orbitalis, pars triangularis* and *pars opercularis*). The opercular part forms the frontal operculum. Occasionally, the *diagonal sulcus* (*sulcus diagonalis* of Eberstaller) can divide the opercular part of the inferior frontal gyrus into two parts. The triangular part may also be indented from above by a *radiate sulcus* (*sulcus radiatus* of Eberstaller). The orbital part is continuous with the basal surface of the frontal lobe, where it merges with the lateral orbital gyrus. The triangular and opercular parts form together the *motor language area* of Broca (1863); Amunts et al. (1999); Amunts and Zilles (2012). Recent mapping approaches based on cytoarchitecture, transmitter receptor distribution and connectivity revealed a highly differentiated segregation of this region (Amunts and Zilles, 2012). The *frontomarginal sulcus* (*sulcus frontomarginalis* of Wernicke) is fairly constant, found at the frontal pole, and connected posteriorly with the middle frontal sulcus. It has two branches, one deep medial branch that borders the frontopolar gyri, and a shallow lateral branch that separates the frontomarginal sulcus from the medial frontal gyrus and the orbital part of the inferior frontal gyrus, respectively. The *frontopolar area* (*area frontopolaris*) at the *frontal pole* (*polus frontalis*) shows three frontopolar gyri, superior, middle, and inferior, that are clearly separated by limiting sulci, interposed between the superior frontal gyrus and the frontomarginal gyrus. Bludau et al. (2014) distinguished two cytoarchitectonically and functionally distinct areas: the lateral frontopolar area 1 (Fp1) and the medial frontopolar area 2 (Fp2).

The **temporal lobe** (**lobus temporalis**) is formed by the *superior, middle* and *inferior temporal gyri* (*gyrus temporalis superior*, -*medius*, and - *inferior*), classically numbered T1, T2, and T3, separated by the *superior* and *inferior temporal sulci* (*sulcus temporalis superior* and *- inferior*, classically numbered t1 and t2). The *temporopolar cortex* (*cortex temporopolaris*) at the *temporal pole* (*polus temporalis*) is a heterogenous region, situated between isocortex laterally, proisocortex in caudorostral continuation and paleocortex caudodorsally (Ding et al., 2009; Blaizot et al., 2010).

**Figure 7:**
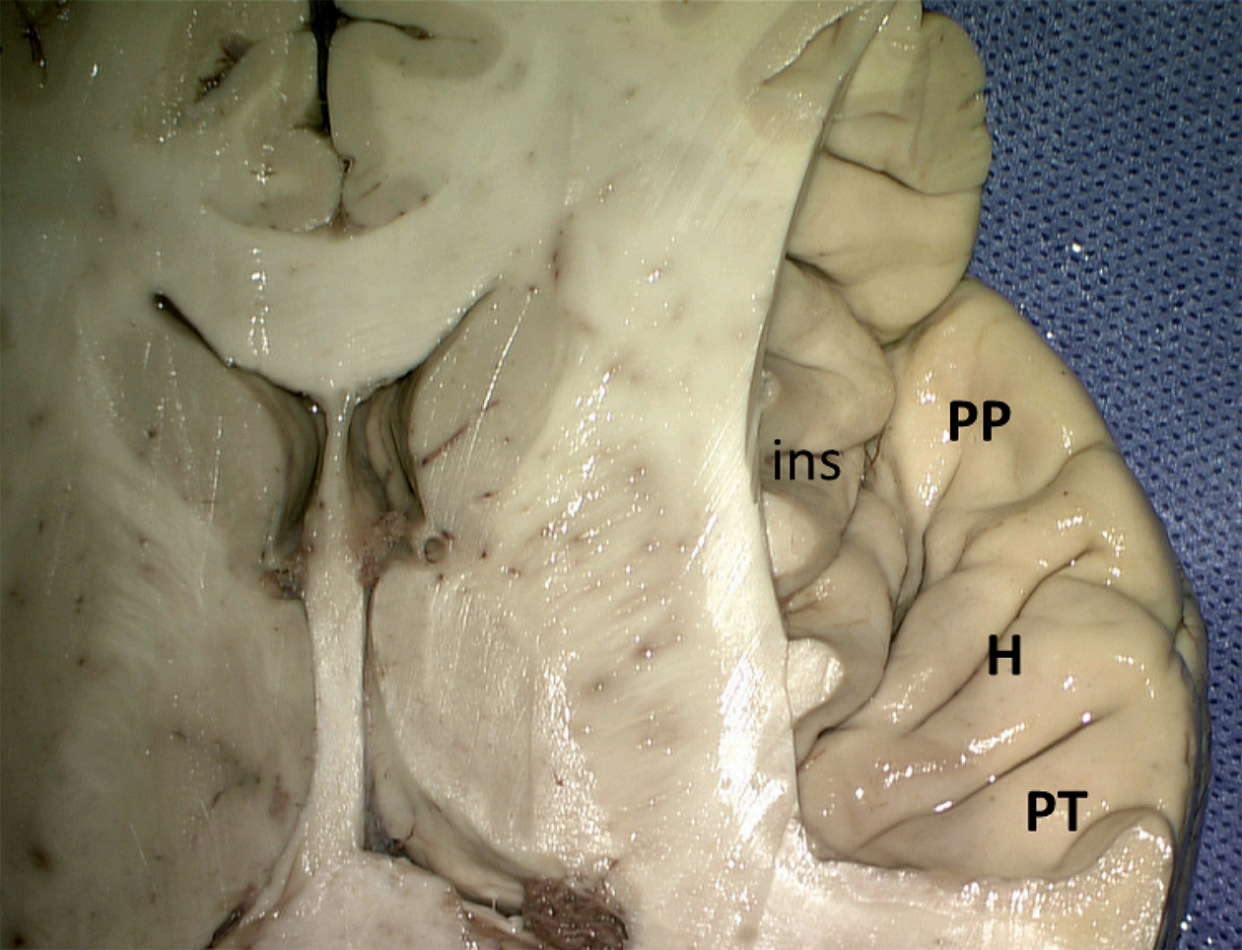
Superior view of the right superior temporal sulcus (kindly provided by Robert Bartoš, Ústí nad Labem, Czech Republic). *ins*, insula; *H*, Heschl gyri; *PP*, planum polare; *PT*, planum temporale.

On the upper surface of the superior temporal gyrus (Figure 7), forming the temporal operculum, the *planum polare*, the *anterior* and *posterior transverse gyri* (*gyrus temporalis transversus anterior* and *- posterior* of Heschl) and the *planum temporale* can be distinguished, separated by sulci. The *anterior transverse temporal sulcus* (*sulcus temporalis transversus anterior*) separates the planum polare from the transverse temporal gyri of Heschl, the two transverse temporal gyri are subdivided by the *intermediate transverse temporal sulcus* (*sulcus temporalis transversus intermedius*), and the *posterior transverse temporal sulcus* (*sulcus temporalis transversus posterior*) separates the posterior transverse temporal gyrus from the planum temporale. There is usually one transverse gyrus of Heschl on the left and two on the right (Heschl, 1878; Marie et al., 2015; Tzourio-Mazoyer and Mazoyer, 2017). These transverse gyri contain the primary auditory cortex. The *planum temporale* is on the left usually larger than on the right (von Economo and Horn, 1930; Geschwind and Levitsky, 1968; Galaburda et al., 1978; Ide et al., 1999; Tzourio-Mazoyer and Mazoyer, 2017). The posterior part of the superior temporal gyrus forms the *sensory* or *receptive language area* of Wernicke (1874).

The temporal lobe is the location of strong asymmetries of its surface with a strong leftward asymmetry of the planum temporale (von Economo and Horn, 1930; Geschwind and Levitsky, 1968; Galaburda et al., 1978; Ide et al., 1999; Toga and Thompson, 2003; Tzourio-Mazoyer and Mazoyer, 2017), the Heschl gyrus and of its sulci depth. A leftward asymmetry of the lateral sulcus is already present at birth (Hill et al., 2010).

**Figure 8:**
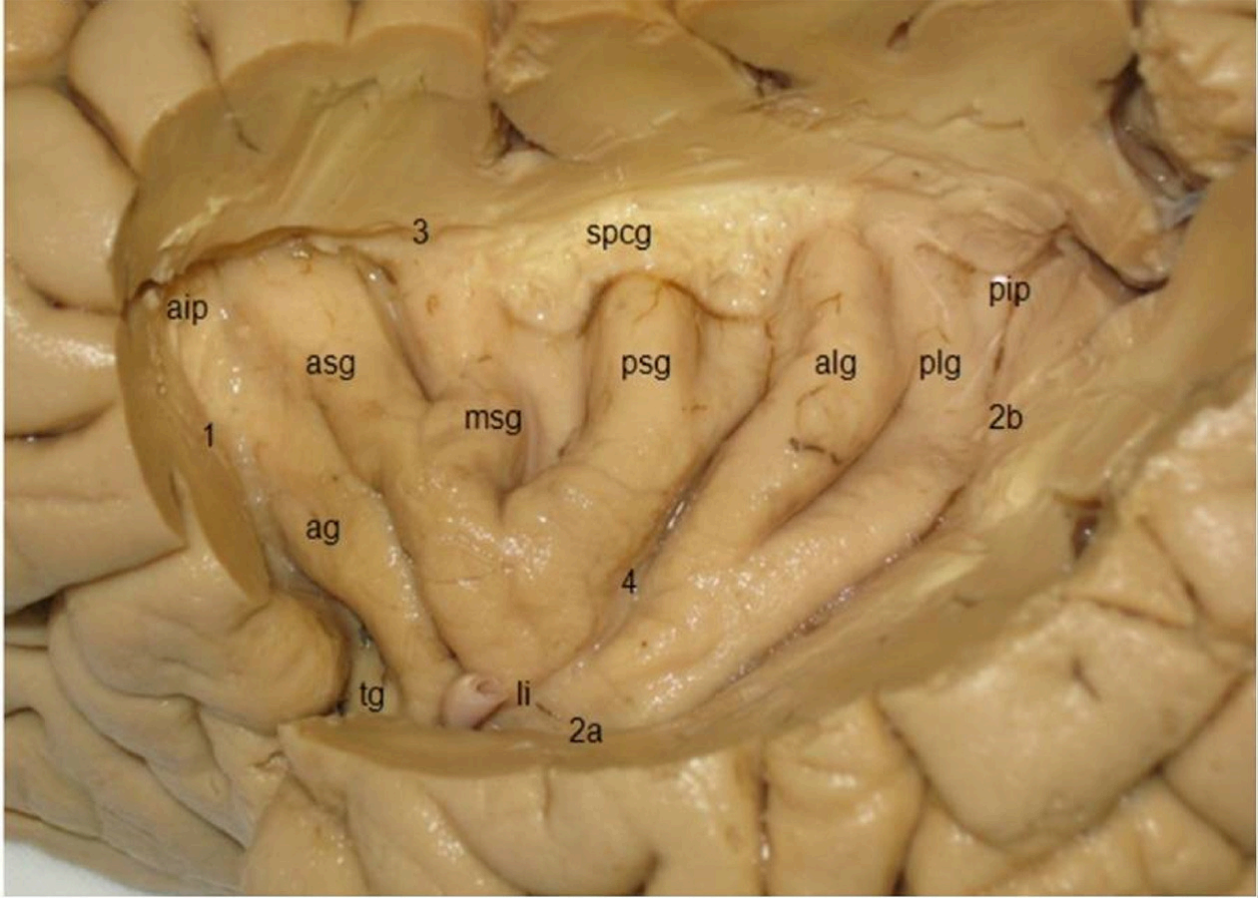
Lateral view of the insula, showing three short gyri, an accessory gyrus and two long gyri (kindly provided by Robert Bartoš, Ústí nad Labem, Czech Republic). *ag*, accessory gyrus; *aip*, anterior insular point; *alg*, anterior long gyrus; *asg*, anterior short gyrus, li limen insulae; *msg*, middle short gyrus; *pip*, posterior insular point; *plg*, posterior long gyrus; *psg*, posterior short gyrus; *spcg*, gyrus supracentralis; *tg*, transverse insular gyrus; *1*–*3*, circular insular sulcus (*1* anterior periinsular sulcus, *2a, 2b*, inferior insular sulcus, horizontal, and posterior parts; *3*, superior periinsular sulcus); *4*, central insular sulcus.

The triangular **insula** of Reil lies in the depths of the lateral sulcus and is covered by the frontal, frontoparietal, parietal, and temporal opercula (Türe et al., 1999; Naidich et al., 2004; Morel et al., 2013; Figure 8). The *limen insulae*, the insular threshold or frontotemporal junction, forms the transition from the anterior perforated substance on the basal aspect of the frontal lobe to the insula. The insula is surrounded by the *circular sulcus of the insula* (*sulcus circularis insulae* of Reil) or *periinsular sulcus*, and contains several vertically directed gyri, usually three *short gyri* (*gyri breves insulae*), anterior, middle and posterior, and one or two *long gyri* (*gyri longi insulae*), anterior and posterior, separated by the *central sulcus of the insula* (*sulcus centralis insulae*) or *transverse insular sulcus* of Eberstaller. The three short gyri converge to the apex of the insula, and are joined to the orbital part of the inferior frontal gyrus by a short annectant gyrus, the *transverse insular gyrus* (*gyrus transversus insulae* of Eberstaller).

The lateral aspect of the **parietal lobe** (**lobus parietalis**) shows the *postcentral gyrus (gyrus postcentralis)*, the *postcentral sulcus (sulcus postcentralis)*, and the *superior* and *inferior parietal lobules (lobulus parietalis superior* and - *inferior)*, numbered P1 and P2, respectively, and separated by the *intraparietal sulcus (sulcus intraparietalis)*. Dorsally, the parietal lobe is connected with the occipital lobe by the *parietooccipital arc (arcus parietooccipitalis)* of Gratiolet. Another “pli de passage” connects the posterior part of the angular gyrus with the superior occipital gyrus. In monkeys, the intraparietal sulcus contains numerous **intraparietal areas** (AIP, LIP, MIP, PIP, and VIP; Rizzolatti et al., 1998; ten Donkelaar, 2011; Zilles and Amunts, 2012). In an fMRI study, Seitz and Binkofski (2003) identified AIP and VIP in the human brain. Two cytoarchitectonic areas were identified and termed hIP (human IntraParietal) 1 and hIP2 in the anterior part of the intraparietal sulcus (Choi et al., 2006), which may be the anatomical correlates of VIP and AIP, respectively (see also Zlatkina and Petrides, 2014). A third intraparietal area, hIP3, was delineated in the anterior medial wall of the intraparietal sulcus, directly across hIP1 and hIP2 (Scheperjans et al., 2008a,b).

The **inferior parietal lobule** (**IPL**) consists of the *supramarginal* and *angular gyri* (*gyrus supramarginalis* and - *angularis*), both of which can be further subdivided (see Caspers et al., 2012). The *supramarginal gyrus* surrounds the posterior ascending ramus of the lateral sulcus and can be subdivided into five areas. The *angular gyrus* lies around the caudal end of the superior temporal gyrus and is composed of two areas. The *first intermediate sulcus* (*sulcus intermedius primus* of Jensen) may subdivide the inferior parietal lobule into the supramarginal and angular gyri, and the *second intermediate sulcus* (*sulcus intermedius secundus* of Eberstaller) may be found posterior to the Jensen sulcus, dividing the angular gyrus into anterior and posterior parts.

The *transverse parietal sulcus* (*sulcus parietalis transversus* of Brissaud) may subdivide the **superior parietal lobule** (**SPL**) into anterior and posterior portions, when it extends on the superolateral aspect of the cerebrum. The SPL includes the preparietal area, the superior parietal area, each with subdivisions (see Scheperjans et al., 2008a,b). The *parietal operculum* (*operculum parietale*) contains four cytoarchitectonic areas (OP1-OP4), corresponding to the secondary somatosensory cortex (Eickhoff et al., 2006a,b).

Most of the **occipital lobe** (**lobus occipitalis**) is found on the medial aspect of the cerebrum. An imaginary line between the parietooccipital sulcus superiorly and the preoccipital notch inferiorly indicates the border between the occipital lobe and the parietal and temporal lobes. On the superolateral aspect, the following occipital gyri and sulci can be found: the *superior occipital gyrus* (O1 or *gyrus occipitalis superior*), the *middle occipital gyrus* (O2 or *gyrus occipitalis medius*), the upper and lower parts of which are separated by the *lunate sulcus* (*sulcus lunatus*), the *inferior occipital gyrus* (O3 or *gyrus occipitalis inferior*) and the *descending occipital gyrus* (*gyrus occipitalis descendens* of Ecker). An *inferior occipital sulcus* (*sulcus occipitalis inferior*) may divide the lower part of O2 from O3. For variations of the gyri and sulci on the occipital lobe convexity, see Ono et al. (1990), Alves et al. (2012) and Malikovic et al. (2012).

## Inferomedial surface of the cerebral hemisphere

**Figure 9:**
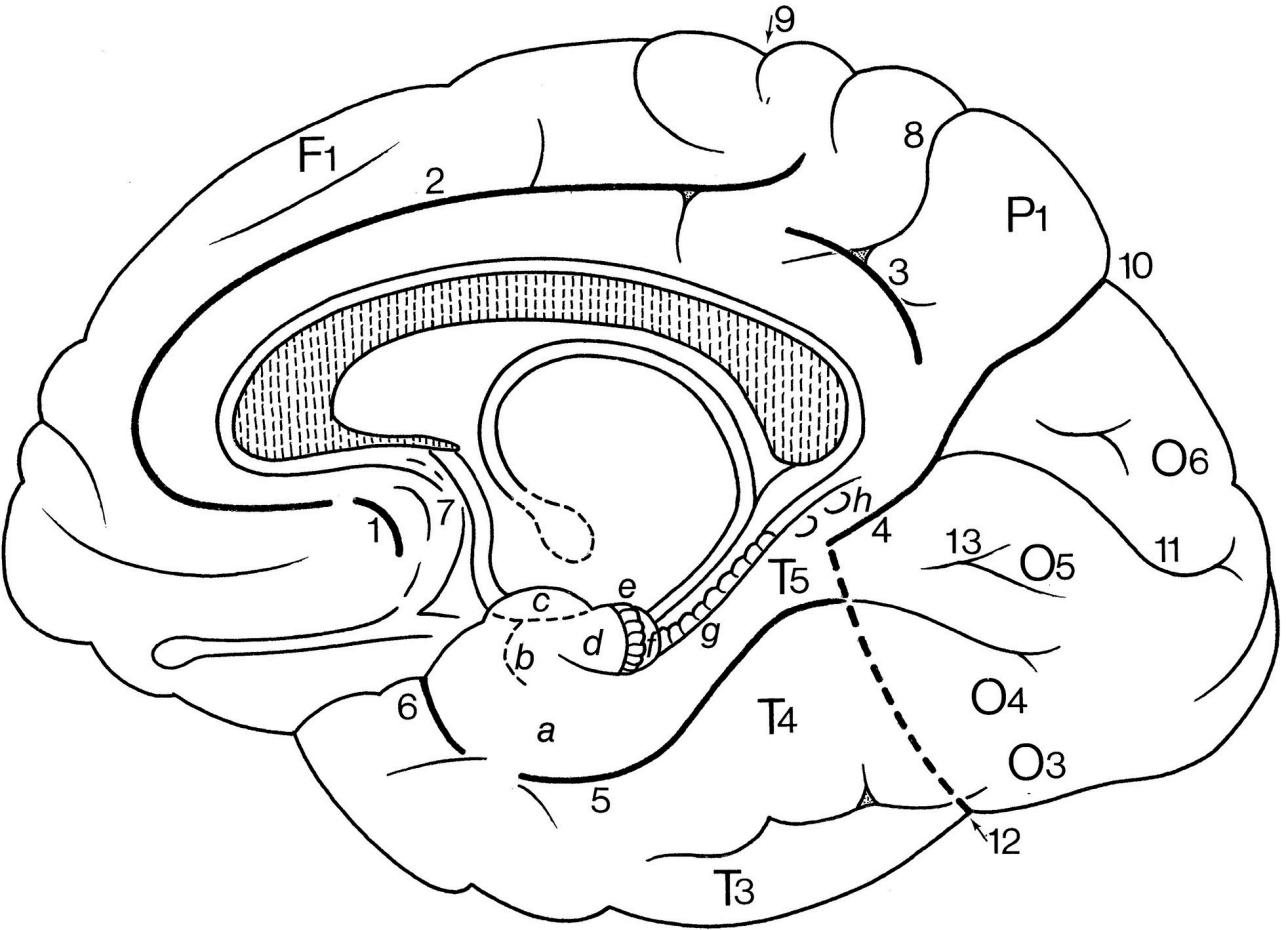
Gyri and sulci on the inferomedial surface of the cerebral hemisphere (after Duvernoy, 1992; ten Donkelaar et al., 2018). (1) below F1, the paracingulate sulcus; and (2) below the subcallosal part of 2, the rostral sulcus. *a*, entorhinal cortex; *b*, ambient gyrus; *c*, semilunar gyrus; *d*, uncinate gyrus*; e*, band of the dentate gyrus of Giacomini; *f*, intralimbic gyrus or uncal apex; *g*, dentate gyrus; *h*, gyri of Andreas Retzius or subsplenial gyri; *F1*, superior frontal gyrus; *P1*, precuneus; *O3*, inferior occipital gyrus; *O4*, fusiform gyrus (occipital part); *O5*, lingual gyrus; *O6*, cuneus; *T3*, inferior temporal gyrus; *T4*, fusiform gyrus (temporal part); *T5*, parahippocampal gyrus; *1*–*6*, parts of the “limbic sulcus” or “scissure limbique”: *1*, anterior paraolfactory sulcus; *2*, cingulate sulcus; *3*, subparietal sulcus; *4*, anterior part of parietooccipital sulcus; *5*, collateral sulcus; *6*, rhinal sulcus; *7*, posterior paraolfactory sulcus; *8*, transverse parietal sulcus of Brissaud; *9*, central sulcus; *10*, parietooccipital sulcus; *11*, calcarine sulcus; *12*, preoccipital notch of Meynert; *13*, lingual sulcus.

On the inferomedial surface of the cerebral hemisphere, **interlobar sulci** include the continuation of the central sulcus, the cingulate sulcus, the sulcus of the corpus callosum, the parietoccipital sulcus, the subparietal sulcus and the collateral sulcus (Figure 9; and Table 2). The *cingulate sulcus* (*sulcus cinguli* or “scissure limbique” of Broca, 1878b) runs parallel to the corpus callosum and ascends above the posterior part (the splenium) of the corpus callosum toward the superior margin of the hemisphere. It gives off a *marginal branch* or *sulcus* (*ramus marginalis* or *sulcus marginalis*). The cingulate sulcus continues around the rostrum of the corpus callosum, where it is also known as the *superior rostral sulcus* (*sulcus rostralis superior*). This sulcus may continue as the *inferior rostral sulcus* (*sulcus rostralis inferior*), which separates the straight gyrus from the medial surface of the frontal lobe (see Figure 5). Immediately rostral to the ascending part of the cingulate sulcus courses the medial end of the central sulcus. The cingulate sulcus divides the medial aspect of the cerebral cortex into an outer and an inner zone. The **outer zone** is composed of the medial part of the *superior frontal gyrus* (F1 or *gyrus frontalis superior*) and the *paracentral lobule* (*lobulus paracentralis*), which surrounds the medial end of the central sulcus, and has frontal and parietal components. Frequently, a series of furrows delineates the *paracingulate sulcus* (*sulcus paracinguli*), which separates the medial division of the superior frontal gyrus from the *paracingulate gyrus* (*gyrus paracinguli*; see Figure 2B), also known as the external cingulate gyrus (Ono et al., 1990). This gyrus is separated ventrally by the cingulate sulcus from the cingulate gyrus (Ono et al., 1990; Paus et al., 1996; Ide et al., 1999). Such a double-parallel pattern, where the paracingulate sulcus surrounds the cingulate sulcus, was found in 24% of either hemisphere in Ono's cases. Ide et al. (1999) found a single sulcus more frequently on the right (69%) than on the left (31%) hemispheres, whereas the double pattern was more frequent on the left (68%) than right (32%) hemispheres.

**Table 2:** Sulci and gyri on the inferomedial surface of the cerebral hemisphere (based on TNA, 2017; ten Donkelaar et al., 2018).

**English official terms and synonyms**	**Latin official terms and synonyms**	**Abbreviations and acronyms**	**Eponyms**
**Inferomedial interlobar sulci**	**Sulci Interlobares inferomediales**	
sulcus of corpus callosum	sulcus corporis callosi	scc
cingulate sulcus	sulcus cinguli	cgs
marginal branch; marginal sulcus	ramus marginalis; sulcus marginalis	cgsmb
parietooccipital sulcus	sulcus parietooccipitalis	pos	sulcus of Gratiolet
subparietal sulcus	sulcus subparietalis	sps
collateral sulcus	sulcus collateralis	cos
central sulcus	sulcus centralis	ces
**Frontal lobe**	**Lobus frontalis**	
inferomedial superior frontal gyrus	gyrus frontalis superior inferomedialis	SFGM; F1
paracingulate sulcus	sulcus paracinguli	pcgs
paracingulate gyrus	gyrus paracinguli	PCG
paracentral sulcus	sulcus paracentralis	pacs
paracentral lobule	lobulus paracentralis	PCL
anterior paracentral gyrus	gyrus paracentralis anterior	APaG
subcallosal area; subcallosal gyrus	area subcallosa; gyrus subcallosus	SCA
paraterminal gyrus	gyrus paraterminalis	PTG
paraolfactory area	area paraolfactoria	PaOA
paraolfactory gyrus	gyrus paraolfactorius	PaOG
paraolfactory sulci	sulci paraolfactorii	
anterior paraolfactory sulcus	sulcus paraolfactorius anterius	apaos
posterior paraolfactory sulcus	sulcus paraolfactorius posterius	ppaos
orbital gyri	gyri orbitales	
medial orbital gyrus	gyrus orbitalis medialis	MOrG
anterior orbital gyrus	gyrus orbitalis anterior	AOrG
posterior orbital gyrus	gyrus orbitalis posterior	POrG
lateral orbital gyrus	gyrus orbitalis lateralis	LOrG
posteromedial orbital lobule	lobulus orbitalis posteromedialis	PMOL
Posterolateral orbital region	regio orbitalis posterolateralis	PLOR
orbital sulci	sulci orbitales	
lateral orbital sulcus	sulcus orbitalis lateralis	lors
transverse orbital sulcus	sulcus orbitalis transversus	tors
medial orbital sulcus	sulcus orbitalis medialis	mors
superior rostral sulcus	sulcus rostralis superior	srs
inferior rostral sulcus	sulcus rostralis inferior	irs
straight gyrus	gyrus rectus	SG
olfactory sulcus	sulcus olfactorius	ols
anterior perforated substance; rostral perforated substance	substantia perforata anterior; substantia perforata rostralis	APS
**Olfactory structures**	**Structurae olfactoriae**	
olfactory bulb	bulbus olfactorius	OB
olfactory peduncle	pedunculus olfactorius	op
olfactory tract	tractus olfactorius	ot
olfactory trigone	trigonum olfactorium	OT
olfactory tubercle	tuberculum olfactorium	Tu
olfactory striae	striae olfactoriae	
medial olfactory stria	stria olfactoria medialis	mos
lateral olfactory stria	stria olfactoria lateralis	los
retrobulbar region	regio retrobulbaris	RBR
piriform cortex	cortex piriformis; cortex olfactorius primarius	Pir
frontal part	pars frontalis	PirF
temporal part	pars temporalis	PirT
**Parietal lobe**	**Lobus parietalis**	
paracentral lobule	lobulus paracentralis	PCL
posterior paracentral gyrus	gyrus paracentralis posterior	PPaG
transverse parietal sulcus	sulcus parietalis transversus	tps	sulcus of Brissaud
precuneus	precuneus	PCun; P1
subparietal sulcus	sulcus subparietalis	sps
**Occipital lobe**	**Lobus occipitalis**	
cuneus	cuneus	Cun; O6
calcarine sulcus	sulcus calcarinus	cas
lingual gyrus; medial occipitotemporal gyrus	gyrus lingualis; gyrus occipitotrmporalis medialis	LG; O5
fusiform gyrus; lateral occipitotemporal gyrus	gyrus fusiformis; gyrus occipitotemporalis lateralis	FG; O4
occipitotemporal sulcus; lateral occipitotemporal sulcus	sulcus occipitotemporalis; sulcus occipitotemporalis lateralis	ots
**Temporal lobe**	**Lobus temporalis**	
inferomedial inferior temporal gyrus	gyrus temporalis inferior inferomedialis	ITGM; T3
occipitotemporal sulcus; lateral occipitotemporal sulcus	sulcus occipitotemporalis; sulcus occipitotemporalis lateralis	ots
fusiform gyrus; lateral occipitotemporal gyrus	gyrus fusiformis; gyrus occipitotemporalis lateralis	FG; T4
medial part	pars medialis	FGM
lateral part	pars lateralis	FGL
ectorhinal cortex	cortex ectorhinalis	EcC
midfusiform sulcus	sulcus fusiformis medius	mfs
collateral sulcus; medial occipitotemporal sulcus	sulcus collateralis; sulcus occipitotemporalis medialis	cos
parahippocampal gyrus	gyrus parahippocampalis	PHG; T5

**Figure 10:**
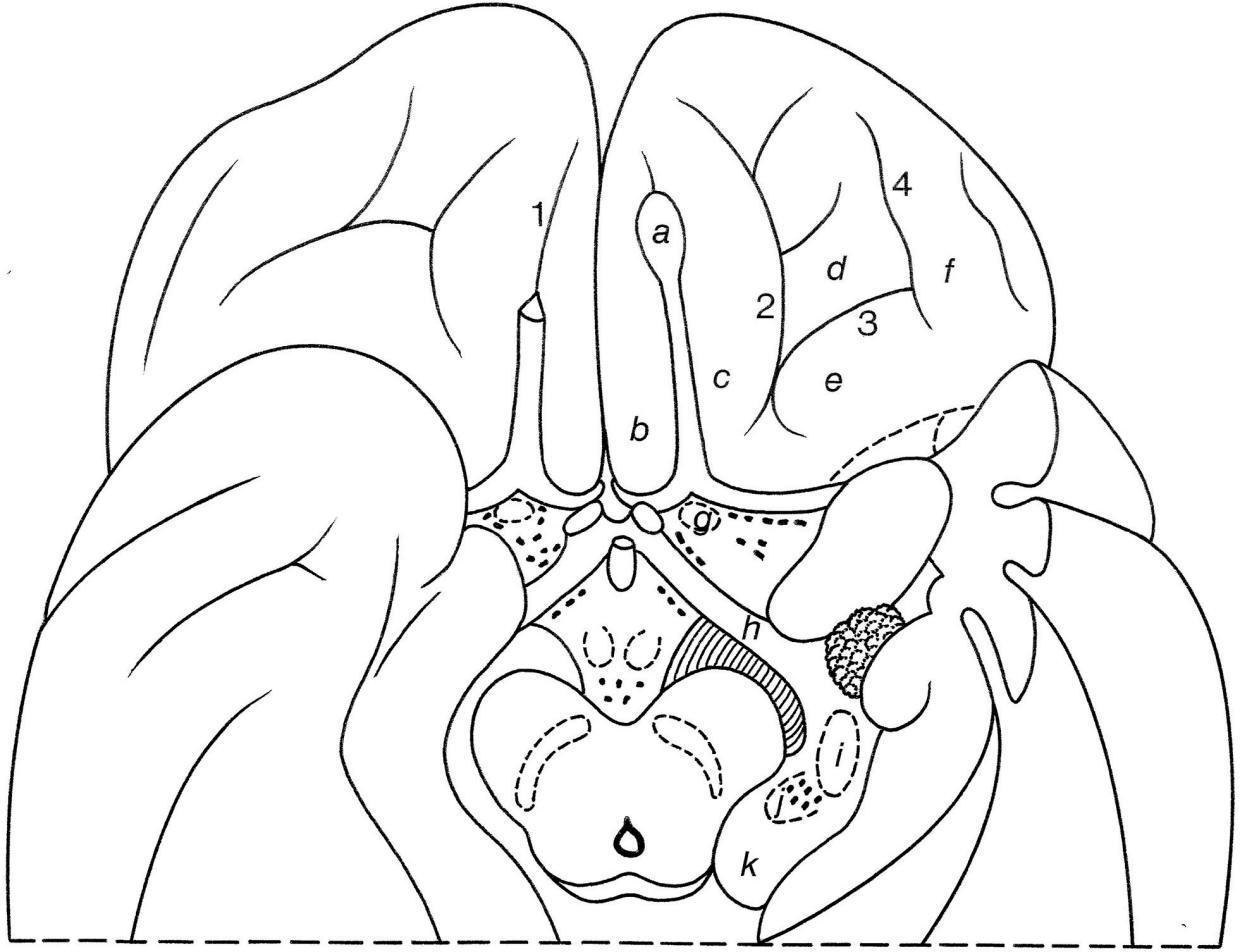
Gyri and sulci on the orbital part of the frontal lobe, shown for the basal surface of the cerebral hemisphere (after Duvernoy, 1992; ten Donkelaar et al., 2018). *a*, olfactory bulb; *b*, straight gyrus; *c, d, e, f*, medial, anterior, posterior, and lateral olfactory gyri; *g*, olfactory tubercle; *h*, optic tract; *i*, lateral geniculate body; *j*, medial geniculate body; *k*, pulvinar; *1*, olfactory sulcus; *2*, medial orbital sulcus; *3*, transverse orbital sulcus; *4*, lateral orbital sulcus.

*For summarizing figures, see Figures 9, 10*.

Caudal to the paracentral lobule lies the large *precuneus* (P1), bordered by the *marginal branch of the cingulate sulcus (ramus marginalis sulci cinguli)* rostrally, the *parietooccipital sulcus (sulcus parietooccipitalis* of Gratiolet) caudally, and the *subparietal sulcus (sulcus subparietalis)* ventrally.

The **inner zone**, separated from the corpus callosum by the *sulcus of the corpus callosum* (*sulcus corporis callosi*), and earlier known as the *fornicate gyrus* (*gyrus fornicatus* of Meynert), is formed by the *cingulate gyrus* (*gyrus cinguli*). The cingulate gyrus can be divided into four parts: an anterior part, a midcingulate cortex, a posterior part and a retrosplenial part (Vogt and Palomero-Gallagher, 2012). The cingulate gyrus is continuous through a narrowing (*isthmus gyri cinguli*) with the *parahippocampal gyrus* (*gyrus parahippocampalis* or T5) in the temporal lobe. The *collateral sulcus* (*sulcus collateralis*, also known as the medial occipitotemporal sulcus) separates T5 from T4, the temporal part of the *fusiform gyrus* (*gyrus fusiformis*, also known as the lateral occipitotemporal gyrus). Areas of the fusiform gyrus within the inferotemporal cortex are part of the ventral visual stream area (see Rosenke et al., 2018), and they process higher-order visual information associated with faces, limbs, words, and places. Caspers et al. (2013) identified two areas, FG1 and FG2, medial and lateral in the posterior part of the fusiform gyrus, respectively. Lorenz et al. (2017) identified two new areas, FG3 and FG4, medial and lateral in the midfusiform gyrus, respectively, separated by the *midfusiform sulcus* (*sulcus fusiformis medius*). The *occipitotemporal sulcus* (*sulcus occipitotemporalis*, also known as the lateral occipitotemporal sulcus) separates the medial part of the *inferior temporal gyrus* (T3 or *gyrus temporalis inferior*) from T4. Various classifications for the temporal sulci and gyri have been published (Ono et al., 1990; Duvernoy, 1992; Hanke, 1997; Huntgeburth and Petrides, 2012; Chau et al., 2014; Cikla et al., 2016) with different relations between the collateral and rhinal sulci and patterns of the various sulci.

The posterior part of the medial cerebral cortex has two deep sulci, which converge toward the splenium. The interlobar *parietooccipital sulcus* (*sulcus parietooccipitalis* of Gratiolet) separates the parietal and occipital lobes, and the lobar *calcarine sulcus* (*sulcus calcarinus*) divides the **occipital lobe** into a dorsal part, the *cuneus* (O6) and a ventral part, the *lingual* or *medial occipitotemporal gyrus* (O5; *gyrus lingualis* or *gyrus occipitotemporalis medialis*). The lingual gyrus may be divided into two parts by the *lingual sulcus* (*sulcus lingualis*). The primary visual cortex is mainly found on both sides of the calcarine sulcus. Below the lingual gyrus, separated by the *occipitotemporal sulcus* (*sulcus occipitotemporalis*), lies the occipital part of the *fusiform* or *lateral occipitotemporal gyrus* (O4; *gyrus fusiformis* or *gyrus occipitotemporalis lateralis*). The visual areas outside the *striate area* (*area striata*) are grouped together as the *extrastriate areas* (*areae extrastriatae*; for current views and further discussion, see Wang et al., 2015).

## Basal surface of the cerebral hemisphere

On the basal surface of the cerebral hemisphere, the occipital lobes are largely covered by the cerebellum, so only the frontal and temporal lobes are visible (Figure 10; and Table 2). On the orbital surface of the frontal lobe, the *olfactory sulcus* (*sulcus olfactorius*) with the **olfactory bulb** and **tract** separates the *straight gyrus* (*gyrus rectus*) from the *orbital gyri*. The olfactory tract divides into the **medial** and **lateral olfactory striae**, of which only the lateral olfactory tract contains secondary olfactory fibers. Between these striae lies the **anterior perforated substance** of Vicq d'Azyr, a region studded with small openings through which the anteromedial central arteries and the recurrent artery of Heubner from the anterior cerebral artery and the lenticulostriate arteries from the middle cerebral artery pass to the basal ganglia and the internal capsule. The medial part of the temporal lobe is formed by the *parahippocampal gyrus* (T5; *gyrus parahippocampalis* or *medial occipitotemporal gyrus*), the continuation of the cingulate gyrus. The most rostral part of the parahippocampal gyrus protrudes medially as the **uncus**. Below the uncus lies the **amygdala**. Lateral to the parahippocampal gyrus, the following structures can successively be observed: the *collateral sulcus* (*sulcus collateralis*), the *fusiform gyrus* (T4; *gyrus fusiformis*) or *lateral occipitotemporal gyrus*, the *occipitotemporal sulcus* (*sulcus occipitotemporalis*), and the *inferior temporal gyrus* (T3; *gyrus temporalis inferior*).

The naming of two “olfactory gyri” in the TA (1998) suggested that there were clearly identifiable gyral structures; this is not true. These terms persisted from the old description of the “rhinencephalon” (see Gastaut and Lammers, 1961; Stephan, 1975) and have been deleted in the TNA (2017). The real olfactory cortex is the *piriform* or *primary olfactory cortex* (*cortex piriformis* or *cortex olfactorius primarius*), which can be divided into frontal and temporal parts (Allison, 1954; Heimer et al., 1977, 1999; Zilles, 2004; Zilles and Amunts, 2012; ten Donkelaar et al., 2018).

In the TNA (2017), the TA names for the sulci and gyri in the orbitofrontal cortex have been corrected. Lateral to the olfactory sulcus, there are two longitudinally directed sulci, the *medial orbital sulcus* (*sulcus orbitalis medialis*) and the *lateral orbital sulcus* (*sulcus orbitalis lateralis*), which are joined by the *transverse orbital sulcus* (*sulcus orbitalis transversus*) to form an H or a K pattern (Duvernoy, 1992; Chiavaras and Petrides, 2000; Öngur et al., 2003; Petrides and Pandya, 2012; Rolls et al., 2015; ten Donkelaar et al., 2018). The following orbital gyri can be found: the *medial orbital gyrus* (*gyrus orbitalis medialis*) between the olfactory sulcus and the medial orbital sulcus, the *anterior orbital gyrus* (*gyrus orbitalis anterior*), the cortex rostral to the transverse orbital sulcus, the *posterior orbital gyrus* (*gyrus orbitalis posterior*), the cortex caudal to the transverse orbital sulcus, and the *lateral orbital gyrus* (*gyrus orbitalis lateralis*) lateral to the lateral orbital sulcus. The caudal parts of the medial and posterior orbital gyri merge to form the *posteromedial orbital lobule* (*lobulus orbitalis posteromedialis*) as described by Türe et al. (1999) and Naidich et al. (2004). The posteromedial orbital lobule gives rise to the *transverse insular gyrus* (*gyrus transversus insulae*). Mai and Majtanik (2017) also distinguished a *posterolateral orbital region* (*regio orbitalis posterolateralis*) between the posterior orbital gyrus and the orbital part of the inferior frontal gyrus.

## The limbic lobe

**Figure 11:**
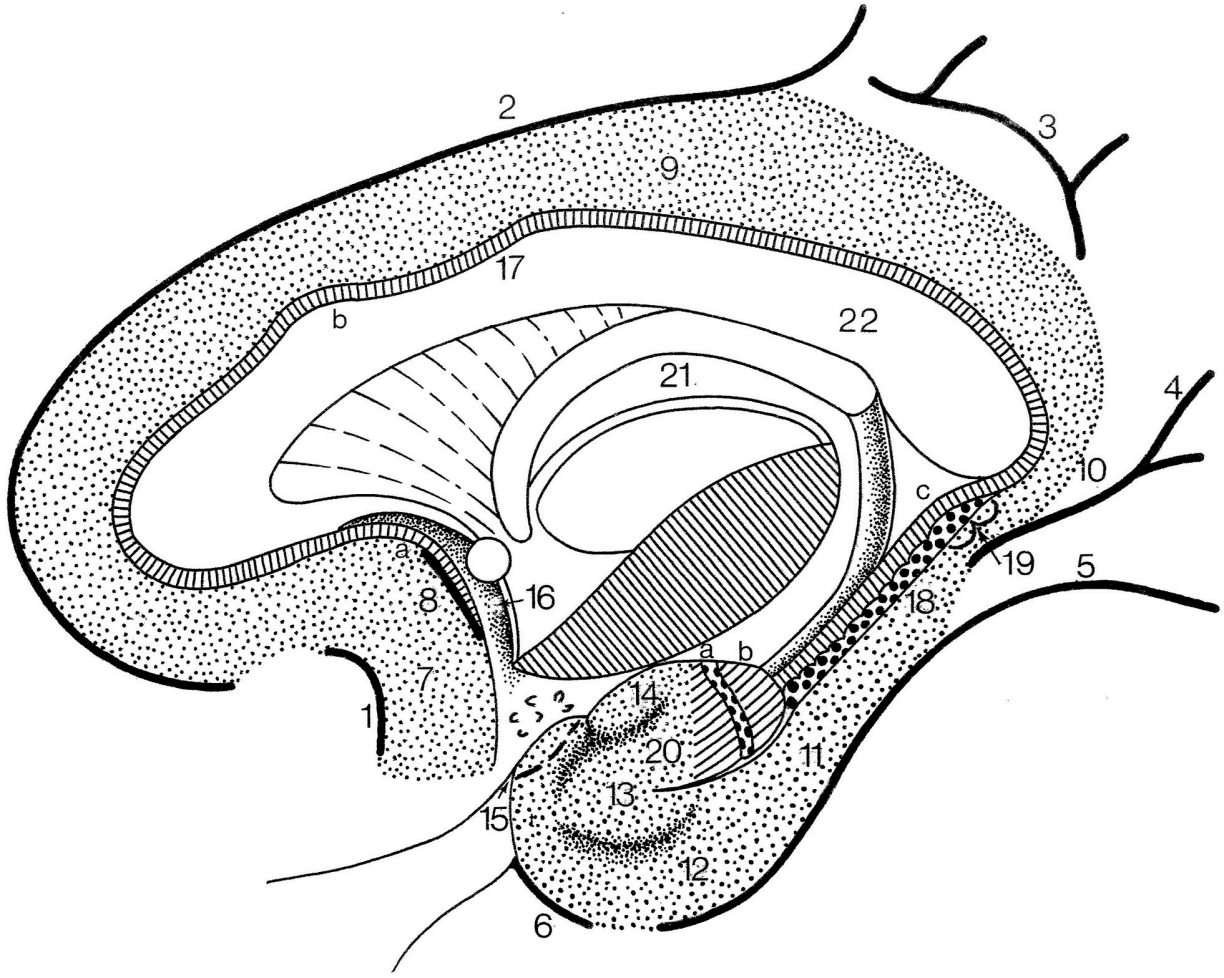
The limbic lobe (after Duvernoy, 1998; ten Donkelaar et al., 2018). *1*, anterior paraolfactory sulcus; *2*, cingulate sulcus; *3*, subparietal sulcus; *4*, rostral part of calcarine sulcus; *5*, collateral sulcus; *6*, rhinal sulcus; *7*, subcallosal gyrus; *8*, posterior paraolfactory sulcus; *9*, cingulate gyrus; *10*, isthmus; *11*, parahippocampal gyrus; *12*, entorhinal cortex; *13*, ambient gyrus; *14*, semilunar gyrus; *15*, piriform cortex; *16*, paraterminal gyrus; *17*, indusium griseum; *18*, dentate gyrus; *19*, gyri of Andreas Retzius; *20*, uncinate gyrus; *21*, fornix; *22*, corpus callosum; *a*, bandelette of Giacomini; *b*, apex of uncus.

The cingulate gyrus and the parahippocampal gyrus form a border (limbus) around the corpus callosum and the brain stem (Broca, 1878b). Broca subdivided his *grand lobe limbique* into inner (the hippocampal formation) and outer (the cingulate and parahippocampal) rings for which now the general descriptive term **limbic lobe** is used (Heimer et al., 2008; Nieuwenhuys et al., 2008). The **“scissure limbique”** separates the limbic lobe from the rest of the cerebral cortex and can be seen as an **interlobar sulcus** (Duvernoy, 1992; ten Donkelaar et al., 2018). It consists of (Figure 11; and Table 3): the *anterior paraolfactory sulcus* (*sulcus paraolfactorius anterior*) in the subcallosal area, the *cingulate sulcus* (*sulcus cinguli*), part of the subparietal sulcus, the rostral part of the parietooccipital sulcus, the *collateral sulcus* (*sulcus collateralis*), and the *rhinal sulcus* (*sulcus rhinalis*).

**Table 3:** Structures of the limbic lobe (based on TNA, 2017; ten Donkelaar et al., 2018).

**English official terms and synonyms**	**Latin official terms and synonyms**	**Abbreviations and acronyms**	**Eponyms**
**Limbic gyrus; outer ring of limbic lobe**	**Gyrus limbicus**	
subcallosal area; subcallosal gyrus	area subcallosa; gyrus subcallosus	SCA
cingulate gyrus	gyrus cinguli	CG
anterior cingulate cortex	gyrus cinguli, pars anterior	ACC
midcingulate cortex	gyrus cinguli, pars media	MCC
posterior cingulate cortex	gyrus cinguli, pars posterior	PCC
retrosplenial cortex	cortex retrosplenialis	RSC
isthmus of cingulate gyrus	isthmus gyri cinguli	ICG
parahippocampal gyrus	gyrus parahippocampalis	PHG; T5
entorhinal cortex	cortex entorhinalis	EC
white reticular substance	substantia reticularis alba		substance of Arnold
hippocampal warts	verrucae hippocampi	
perirhinal cortex	cortex perirhinalis	PRC
uncus	uncus	Un
ambient gyrus	gyrus ambiens	AmG
semianular sulcus	sulcus semianularis	sas
semilunar gyrus	gyrus semilunaris	SLG
uncinate gyrus	gyrus uncinatus	UG
band of dentate gyrus	limbus fasciae dentatae	BDG	band of Giacomini
intralimbic gyrus; uncal apex	gyrus intralimbicus	ILG
collateral sulcus	sulcus collateralis	cos
rhinal sulcus	sulcus rhinalis	rhs
intrarhinal sulcus	sulcus intrarhinalis	irhs
**Hippocampal formation; inner ring of limbic lobe**	**Formatio hippocampi**	
precommissural part of hippocampus	pars precommissuralis hippocampi	HiP
supracommissural part of hippocampus	pars supracommissuralis hippocampi	HiS
lateral longitudinal stria	stria longitudinalis lateralis	lls	taenia tecta; stria of Lancisi
indusium griseum	indusium griseum	IGr
medial longitudinal stria	stria longitudinalis medialis	mls	taenia libera; stria of Lancisi
retrocommissural part of hippocampus; hippocampus proper	pars retrocommissuralis hippocampi; hippocampus proprius	HiR
pes hippocampi; pes of hippocampus	pes hippocampi	PHip
head; anterior segment	caput; pars anterior	HiH
body; middle sement	corpus; pars media	HiB
tail; posterior segment	cauda; pars posterior	HiT
hippocampal sulcus	sulcus hippocampalis	his
dentate gyrus	gyrus dentatus	DG
fimbriodentate sulcus	sulcus fimbriodentatus	fds
fimbria of hippocampus	fimbria hippocampi	FiH
gyri of andreas retzius; subsplenial gyri	dentes subiculi; gyri subspleniales	GAR; SG
fasciolar gyrus	gyrus fasciolaris	FG
fasciola cinerea	fasciola cinerea	FC
subiculum	subiculum	S
presubiculum	presubiculum	PrS
parasubiculum	parasubiculum	PaS

*For summarizing figures, see Figures 9, 11*.

The limbic lobe consists of an inner ring (known as the intralimbic gyrus in the French literature; Testut and Latarjet, 1948), the hippocampal formation (see below), and an outer ring, the limbic gyrus. The **limbic gyrus** (*gyrus limbicus*) includes: (1) the *subcallosal area* (*area subcallosa* or *gyrus subcallosus*), which includes the *paraolfactory gyrus* (*gyrus paraolfactorius*) between the anterior and posterior paraolfactory sulci, and the *paraterminal gyrus* (*gyrus paraterminalis*) just rostral to the lamina terminalis; (2) the *cingulate gyrus* (*gyrus cinguli*); (3) the *isthmus of the cingulate gyrus* (*isthmus gyri cinguli*); (4) the *parahippocampal gyrus* (*gyrus parahippocampalis*); (5) the *entorhinal cortex* (*cortex entorhinalis*); and (6) the *uncus*. In the TNA (2017), the uncus is treated as a structure separate from the parahippocampal gyrus, following Insausti and Amaral (2012). The *entorhinal cortex* (cortex entorhinalis; Braak and Braak, 1992; Insausti et al., 1995, 2017) is located at the rostral part of the parahippocampal gyrus, which includes the *uncus* (*uncus*) and small gyri called the the *uncinate gyrus* (*gyrus uncinatus*), the *ambient gyrus* (*gyrus ambiens*) and the *semilunar gyrus* (*gyrus semilunaris*). The entorhinal cortex corresponds to BA28 and has been subdivided into eight different subfields (Insausti et al., 1995). Adjacent is the perirhinal (Anglo-Saxon terminology) or transentorhinal (German terminology) cortex. The entorhinal cortex can be defined macroscopically by the white reticular matter (*substantia reticularis alba* of Arnold) and the hippocampal warts (*verrucae hippocampi*) described by Retzius (1896) and Klingler (1948). The entorhinal cortex is characterized by a dissecting layer (*lamina dissecans*), separating the external and internal layers, for which Rose (1926) introduced the term schizocortex.

The **uncus** *(uncus)* includes a number of bulges: (1) the *uncinate gyrus (gyrus uncinatus)*, its most rostral part, corresponding to the *amygdalohippocampal transition area (area transitionis amygdalohippocampalis)*; (2) the *band of the dentate gyrus (limbus fasciae dentatae* of Giacomini), the middle part, corresponding to the dentate gyrus; and (3) the *intralimbic gyrus* or *uncal apex (gyrus intralimbicus)*, the most caudal part of the uncal bulge and corresponding to the CA3 field. The dorsal limit of the uncus is rather inconspicuous, but its ventral limit is marked by the *hippocampal sulcus (sulcus hippocampalis)*. The hippocampal sulcus continues rostralwards as the *intrarhinal sulcus (sulcus intrarhinalis)*, forming the ventral limit of the *ambient gyrus (gyrus ambiens)*. The *semianular sulcus (sulcus semianularis)* separates the ambient gyrus from the *semilunar gyrus (gyrus semilunaris)*, which forms the periamygdaloid cortex.

The *perirhinal cortex* (*cortex perirhinalis*) is a periarchicortical structure (Suzuki and Amaral, 1994; Augustinack et al., 2013) around the *perirhinal sulcus* (*sulcus perirhinalis*) and corresponds to the *transentorhinal region* (*regio transentorhinalis*) of Braak and Braak (1992). Its laminar structure is comparable to that of the entorhinal cortex. Adjacent to the perirhinal cortex is the *ectorhinal cortex* (*cortex ectorhinalis*), an isocortical part of the inferior temporal surface, but sometimes included in the perirhinal cortex (Ding and Van Hoesen, 2010).

Classically, the **hippocampal formation** (*formatio hippocampi*) is divided into three, originally adjacent, allocortical areas (Stephan, 1975; Duvernoy, 1998; ten Donkelaar, 2011): (1) the *dentate gyrus* (*gyrus dentatus*); (2) the *hippocampus proper* or *Ammon's horn* (*hippocampus proprius* or *cornu ammonis*); and (3) the *subiculum* (*subiculum*). These three structures are known as the **archicortex**. A small indentation between the fimbria and the molecular layer of the dentate gyrus has been termed the *fimbriodentate sulcus* (*sulcus fimbriodentatus*) by Gastaut and Lammers (1961). Several periallocortical structures, including the entorhinal cortex, the presubiculum and the parasubiculum, all parts of the parahippocampal gyrus, have also been included within the term “hippocampal formation,” since they are closely related and share a common pattern of projections (Insausti and Amaral, 2012). The TNA (2017), however, follows the classic view.

The hippocampal formation develops from the medial pallium, and during the outgrowth of the cerebral hemispheres, first caudalwards and subsequently ventralwards and rostralwards, the *retrocommissural part* of the hippocampus (*pars retrocommissuralis hippocampi*) becomes situated in the temporal lobe (see ten Donkelaar et al., 2014). Rudiments of the *supracommissural part* of the hippocampus (*pars supracommissuralis hippocampi*) can be found above the corpus callosum as the *indusium griseum* (*indusium griseum*), a thin cell layer, flanked by the *lateral longitudinal stria* of Lancisi (*stria longitudinalis lateralis*), also known as the taenia tecti, and the *medial longitudinal stria* of Lancisi (*stria longitudinalis medialis*), also known as the taenia libera. The *precommissural part* of the hippocampus (*pars precommissuralis hippocampi*) disappears.

Macroscopically, the following parts of the hippocampus can be distinguished (Duvernoy, 1998; Insausti and Amaral, 2012; ten Donkelaar et al., 2018): (1) the *pes hippocampi* or *pes of the hippocampus* (*pes hippocampi*) showing the *hippocampal digitations* (*digitationes hippocampi*); (2) the *head* or *anterior segment* (*caput* or *pars anterior*); (3) the *body* or *middle segment* (*corpus* or *pars media*); (4) the *tail* or *posterior segment* (*cauda* or *pars posterior*); and (5) the *gyri of Andreas Retzius* or *subsplenial gyri* (*dentes subiculi* or *gyri subspleniales*) described by Gustav Retzius (Retzius, 1896) in honor of his father Anders Adolf, a series of small bumps marking the caudal limit of the CA1 field. Here, the parahippocampal gyrus meets the retrosplenial region caudally. Two obliquely oriented small gyri are located deep to the gyri of Andreas Retzius. The medial one is the *fasciola cinerea* (*fasciola cinerea*), the visible caudal end of the dentate gyrus as described by Giacomini (1884) and Klingler (1948). The lateral gyrus, corresponding to the caudal end of the CA3 field, is the *fasciolar gyrus* (*gyrus fasciolaris*).

## Conclusions

In this review, an attempt for a common terminology for the cerebral gyri and sulci is presented, largely following the recently published Terminologia Neuroanatomica (TNA, 2017). The differences found in the modern literature mainly concern:

1. The use of the term fissure for certain deep sulci; here, it is advocated to restrict the term fissure to the interhemispheric fissure, and to use the term sulcus for all other grooves;
2. The use of the topographical terms lateral and medial occipitotemporal gyri for the fusiform gyrus and the lingual gyrus, respectively.
3. These terms and some other frequently used terms are placed as synonyms, both in English and Latin in the TNA, and are summarized in Tables 1–3.
4. We suggest a simple system of abbreviations with capitals for gyri and small letters for sulci.
5. In the near future, several new subdivisions will have to be included. The TNA database at the FIPAT websites (www.unifr.ch/ifaa; http://FIPAT.library.dal.ca) will be regularly updated.

## Author contributions

All authors listed have made a substantial, direct and intellectual contribution to the work, and approved it for publication.

### Conflict of interest statement

The authors declare that the research was conducted in the absence of any commercial or financial relationships that could be construed as a potential conflict of interest.
