# Supplementary material for: Toward a Common Terminology for the Gyri and Sulci of the Human Cerebral Cortex
Source: Front Neuroanat. 2018 Nov 19;12:93. doi: 10.3389/fnana.2018.00093 (PMC6252390; doi:10.3389/fnana.2018.00093)
Supplement: Supplementary file 1 [file Table_1.DOCX]

**Supplementary Table 1: Synonyms and eponyms for cerebral gyri (based on data by Kéraval, 1884a, b; Dejerine, 1895; Testut and Latarjet, 1948; and Swanson, 2014)**

| **English official term** | **TNA Latin term** | **French term (from Duvernoy 1992)** | **English, French and German synonyms with sources** | **Latin synonyms with sources** | **Eponyms and Acronyms** |
| --- | --- | --- | --- | --- | --- |
| **Gyri on lateral surface** |  |  |  |  |  |
| **Frontal lobe** |  |  |  |  |  |
| Superior frontal gyrus | Gyrus frontalis superior | Gyrus frontal supérieur | Etage frontale supérieur (Gratiolet); Première circonvolution frontale (Broca); Superofrontal gyrus (Huxley); Superior frontal gyrus (Turner); Erster oder oberer Stirnwindung (Bischoff); Oberer Stirnwulst (Pansch) | Gyrus frontalis superior (Ecker, Pansch) | F1 |
| Middle frontal gyrus | Gyrus frontalis medius | Gyrus frontal moyen | Etage frontale moyen (Gratiolet); Deuxième circonvolution frontale (Broca); Mediofrontal gyrus (Huxley); Middle frontal gyrus (Turner); Zweiter oder mittlerer Stirnwindung (Bischoff); Mittlerer Stirnwulst (Pansch) | Gyrus frontalis medius (Ecker, Pansch) | F2 |
| Inferior frontal gyrus | Gyrus frontalis inferior | Gyrus frontal inférieur | Etage frontal inférieur (Gratiolet); Troisième circonvolution frontale (Broca); Inferofrontal gyrus (Huxley); Inferior frontal gyrus (Turner); Dritter oder unterer Stirnwindung (Bischoff); Unterer Stirnwulst (Pansch) | Gyrus frontalis inderior (Ecker, Pansch) | F3 |
| Orbital part | Pars orbitalis | Pars orbitalis | Cortex frontal ventral |  |  |
| Triangular part | Pars triangularis | Pars triangularis | Aire de Broca, à gauche |  | **Broca** |
| Opercular part | Pars opercularis | Pars opercularis | Aire de Broca, à gauche |  | **Broca** |
| Precentral gyrus | Gyrus precentralis | Gyrus précentral | Premier pli ascendent (Gratiolet); Circonvolution frontale ascendente (Charcot); Circonvolution prérolandique (Broca); Circonvolution centrale antérieure; Antero-parietal gyrus (Huxley); Ascending frontal gyrus (Turner); Vordere Centralwindung (Bischoff); Vorderer Rolandischer Wulst (Pansch) | Gyrus centralis anterior (Ecker, Henle); Gyrus Rolandicus anterior (Pansch) | **Rolando** |
| Subcentral gyrus | Gyrus subcentralis | Gyrus subcentral | Pli de passage fronto-pariétal infèrieur (Gratiolet); Opercule rolandique (Broca) |  |  |
| **Insular lobe** |  |  | Circonvolution de la scissure de Sylvius (Vicq d'Azyr); Lobule de la scissure de Sylvius (Cruveilhier); Stammlappen (Reil, Burdach) | Insula (Reil); Lobus caudicis (Burdach, Pansch) | **Reil** |
| Short gyri | Gyri breves insulae |  |  |  |  |
| Long gyrus | Gyrus longus insulae |  |  |  |  |
| **Parietal lobe** |  |  |  |  |  |
| Subcentral gyrus | Gyrus subcentralis |  |  |  |  |
| Postcentral gyrus | Gyrus postcentralis | Gyrus postcentral | Second pli ascendent (Gratiolet); Circonvolution pariétale ascendante (Charcot); Circonvolution postrolandique (Broca); Circonvolution centrale postérieure; Posteroparietal gyrus (Huxley); Ascending parietal gyrus (Turner); Hintere Centralwindung (Bischoff); Hinterer Rolandischer Wulst (Pansch) | Gyrus centralis posterior (Ecker, Henle); Gyrus Rolandicus posterior (Pansch) |  |
| Superior parietal lobule | Lobus parietalis superior | Gyrus pariétal supérieur | Lobule du deuxième pli ascendent (Gratiolet); Première circonvolution pariétale (Broca); Posteroparietal lobule (Huxley, Turner); Oberer Scheitelbeinlappen (Huschke); Oberer Scheitelwulst (Pansch) | Lobulus parietalis superior (Pansch) | P1 |
| Inferior parietal lobule | Lobus parietalis inferior | Gyrus pariétal inférieur | Unterer Scheitelwulst (Pansch) | Lobulus parietalis inferior (Ecker, Pansch); Gyrus parietalis secundus (Schwalbe) | P2 |
| Supramarginal gyrus | Gyrus supramarginalis | Gyrus supramarginal | Lobule du pli marginal supérieur (Gratiolet); Erster oder vorderer Scheitelbogenwindung (Bischoff); Unterer Scheitelbogen (Meynert) | Lobulus tubaris (Henle) |  |
| Angular gyrus | Gyrus angularis | Gyrus angulaire | Pli courbe (Gratiolet); Angular gyrus (Huxley); Zweiter oder mittlerer Scheitelbogenwindung (Bischoff); Oberer Scheitelbogen (Meynert) | Gyrus angularis (Ecker) |  |
| Parieto-occipital arc | Arcus parieto-occipitalis | Arcus parieto-occipitalis | Premier pli de passage pariéto-occipital (Gratiolet); Première circonvolution de passage (Pozzi); Erster Úbergangsfalte (Eberstaller) |  |  |
|  |  | Second pli de passage pariéto-occipital | Second pli de passage pariéto-occipital (Gratiolet); Deuxième circonvolution de passage (Pozzi); Zweiter Úbergangsfalte (Eberstaller) |  |  |
| **Occipital lobe** |  |  |  |  |  |
| Superior occipital gyrus | Gyrus occipitalis superior | Gyrus occipital supérieur | Pli occipital supérieur (Gratiolet); Première circonvolution occipitale (Pozzi); First external annectant gyrus (Huxley); First bridging annectant or connecting gyrus (Turner); Obere innere Scheitelbogenwindung (Bischoff) | Gyrus occipitalis superior (Pansch) | O1 |
| Middle occipital gyrus | Gyrus occipitalis medius | Gyrus occipital moyen | Pli occipital moyen (Gratiolet); Deuxième circonvolution occipitale (Pozzi); Medio-occipital and second external annectant gyrus (Huxley); Hintere oder dritte Scheitelbogenwindung (Bischoff) | Gyrus occipitalis medius (Pansch) | O2 |
| Inferior occipital gyrus | Gyrus occipitalis inferior | Gyrus occipital inférieur | Pli occipital inférieur (Gratiolet); Troisième circonvolution occipitale (Pozzi) | Gyrus temporo-occipitalis (Ecker); Gyrus occipitalis inferior (Pansch) | O3 |
| Descending occipital gyrus | Gyrus occipitalis descendens | Gyrus descendens |  | Gyrus occipitalis descendens (Ecker) | **Ecker** |
| **Temporal lobe** |  |  |  |  |  |
| Superior temporal gyrus | Gyrus temporalis superior | Gyrus temporal supérieur | Pli marginal postérieur et inférieur (Gratiolet); Première circonvolution temporale (Broca); Temporale supérieur (Pozzi); Antero-temporal gyrus (Huxley); Superior temporosphenoidal convolution (Turner); Erste oder aüssere obere Schläfenwindung (Bischoff); Oberer Schläfenwulst (Pansch) | Gyrus temporalis superior sive inframarginalis (Huschke); Gyrus temporalis superior (Ecker, Pansch) | T1 |
| Polar plane | Planum polare |  |  |  | PP |
| Transverse temporal gyri | Gyri temporales transversi | Gyrus temporaux transverses | Circonvolutions temporales transverses; Quere Schläfenwindungen (Heschl) |  | **Heschl** |
| Anterior transverse temporal gyrus | Gyrus temporalis transversus anterior |  | Vordere quere Schläfenwindung (Heschl) |  | **Heschl** |
| Posterior transverse temporal gyrus | Gyrus temporalis transversus posterior |  | Hintere quere Schläfenwindung (Heschl) |  | **Heschl** |
| Temporal plane | Planum temporale |  | Schläfenfläche (Heschl) |  | PT |
| Middle temporal gyrus | Gyrus temporalis medius | Gyrus temporal moyen | Pli temporal moyen et partie descendante du pli courbe (Gratiolet); Deuxième circonvolution temporale (Broca); Temporale inférieur (Pozzi); Medio-temporal gyrus (Huxley); Mittlerer Schläfenwulst (Pansch) | Gyrus temporalis medius (Ecker, Pansch) | T2 |
| Inferior temporal gyrus | Gyrus temporalis inferior | Gyrus temporal inférieur | Pli temporal inférieur (Gratiolet); Troisième circonvolution temporale (Broca); Inferior temporo-sphenoidal gyrus (Turner); Unterer Schläfenwulst (Pansch) | Gyrus temporalis inferior (Ecker, Pansch) | T3 |
|  |  |  |  |  |  |
| **Gyri on inferomedial surface** |  |  |  |  |  |
| **Frontal lobe** |  |  |  |  |  |
| Superior frontal gyrus | Gyrus frontalis superior | Face médiale du gyrus frontal supérieur | Second pli de passage ou la zone externe du lobe fronto-pariétal (Gratiolet); Marginal gyrus (Turner); Periphere Randwulst der Zwinge (Burdach); Erster oder vorderer medialer Primär- oder Hauptwulst (Pansch) | Gyrus medialis fronto-parietalis (Pansch); Gyrus frontalis superior sive primus (Schwalbe) | F1 |
| Paracentral lobule | Lobulus paracentralis | Lobule paracentral | Pli de passage fronto-parietal supérieur (Gratiolet) | Lobulus paracentralis |  |
| Anterior paracentral gyrus | Gyrus paracentralis anterior |  |  |  |  |
| Subcallosal area | Area subcallosa | Gyrus subcalleux |  |  |  |
| Paraterminal gyrus | Gyrus paraterminalis |  |  |  |  |
| Paraolfactory area | Area paraolfactoria |  |  |  |  |
| Paraolfactory gyrus | Gyrus paraolfactorius |  |  |  |  |
| Orbital gyri | Gyri orbitales | Gyri orbitaires | Circonvolutions orbitaires |  |  |
| Medial orbital gyrus | Gyrus orbitalis medialis | Gyrus orbital médial | Circonvolutionolfactive externe (Gerdy); Deuxième circonvolution orbitaire (Broca); Internal and external gyri of orbital lobule (Turner); Mediale orbitale Primär- oder Hauptwulst (Pansch) | Gyrus orbitalis medialis (Pansch) |  |
| Anterior orbital gyrus | Gyrus orbitalis anterior | Gyrus orbital antérieur |  |  |  |
| Posterior orbital gyrus | Gyrus orbitalis posterior | Gyrus orbital postérieur |  |  |  |
| Lateral orbital gyrus | Gyrus orbitalis lateralis | Gyrus orbital latéral | Laterale orbitale Primär- oder Hauptwulst (Pansch) | Gyrus orbitalis lateralis (Pansch) |  |
| Straight gyrus | Gyrus rectus | Gyrus rectus | Circonvolution olfactive interne (Gerdy); Première circonvolution orbitaire (Broca); Anterior part of marginal gyrus (Turner) | Gyrus rectus (Valentin); Gyrus orbitalis medialis (Pansch) |  |
| **Limbic lobe** |  |  |  |  |  |
| Subcallosal area | Area subcallosa | Gyrus subcalleux |  |  |  |
| Cingulate gyrus | Gyrus cinguli | Gyrus cingulaire | Circonvolution du corps calleux (Broca); Circonvolution de l'ourlet (Foville); Pli du corps calleux, grand pli commissural interne (Pozzi); Circpnvolution of corpus callosum (Turner); Callosal gyrus (Huxley); Zwinge, Cingula (Burdach); Bogenwulst, Āusseres Gewölbe (Arnold) | Gyrus cinguli (Burdach, Pansch); Fornix periphericus (Arnold); Gyrus fornicatus (Ecker) |  |
| Isthmus of gyrus cinguli | Isthmus gyri cinguli | Isthme | Plis de passage internes (Pozzi); Pli de passage cunéolimbique (Broca); Zwickelwindung (Ecker); Untere oder fünfte Scheitelbogenwindung (Bischoff) | Gyrus cunei (Ecker); Isthmus gyri fornicati (Broca) |  |
| Parahippocampal gyrus | Gyrus parahippocampalis | Gyrus parahippocampal | Circonvolution à crochet (Vicq d'Azyr); Circonvolution du grand hippocampe (Gratiolet); Hippocampal gyrus (Huxley) | Gyrus hippocampi (Burdach, Henle); Gyrus occipitotemporalis mediales (Ecker, Pansch) |  |
| Entorhinal cortex | Cortex entorhinalis | Aire entorhinale |  |  |  |
| Uncus | Uncus | Uncus |  | Gyrus uncinatus (Ecker) |  |
| Ambient gyrus | Gyrus ambiens | Gyrus ambiens | Inselschwelle (Schwalbe) | Limen fissurae Sylvii (Reil); Limen insulae |  |
| Semilunar gyrus | Gyrus semilunaris | Gyrus semilunaire |  |  |  |
| Uncinate gyrus | Gyrus uncinatus | Gyrus uncinatus |  |  |  |
| Band of dentate gyrus | Limbus fasciae dentatae | Bandelette de Giacomini |  |  | **Giacomini** |
| Intralimbic gyrus | Gyrus intralimbicus | Apex de l'uncus | Uncal apex |  |  |
| Dentate gyrus | Gyrus dentatus | Gyrus dentatus | Corps godronné corps denté (Vicq d'Azyr); Circonvolution godronné (Duval); Dentate gyrus (Huxley) | Substantia cinerea (Tarin); Fascia dentata (Arnold); Gyrus dentatus sive fascia dentata (Ecker) | **Tarin** |
| **Parietal lobe** |  |  |  |  |  |
| Paracentral lobule | Lobulus paracentralis | Lobule paracentral |  |  |  |
| Posterior paracentral gyrus | Gyrus paracentralis posterior |  |  |  |  |
| Precuneus | Precuneus | Face médiale du gyrus pariétal supérieur; Précunéus | Avant-coin; Lobe carré (Gratiolet); Lobule quadrilatère (Foville); Première circonvolution pariétale (Broca); Quadrate lobule (Huxley); Vorzwickel (Burdach, Henle); Oberer Scheitelwulst (Pansch) | Precuneus (Burdach, Ecker); Lobus quadratus (Huxley) |  |
| **Occipital lobe** |  |  |  |  |  |
| Cuneus | Cuneus | Cunéus | Coin; Lobe triangulaire (Broca); Occipital lobule (Turner); Zwickel (Burdach, Henle); Āussere obere Hinterhauptswindung (Bischoff); Oberer Zwischenscheitellappen (Huschke) | Cuneus (Burdach, Ecker); Lobulus medialis posterior (Pansch) | O6 |
| Lingual gyrus | Gyrus lingualis | Gyrus lingual | Deuxième circonvolution temporo-occipitale; Lobule lingual; Medial occipitotemporal gyrus (Turner); Untere innere Hinterhauptswindungszug (Bischoff) | Gyrus occipitotemporalis medialis (Pansch); Lobulus lingualis (Huschke) | O5 |
| Fusiform gyrus | Gyrus fusiformis | Gyrus fusiforme | Première circonvolution temporo-occipitale; Lobule fusiforme; Lateral occipitotemporal gyrus (Turner); Unteräusserer Hinterhauptsbindungszug (Bischoff) | Gyrus occipitotemporalis lateralis (Pansch); Lobulus fusiformis (Huschke) | O4 |
| **Temporal lobe** |  |  |  |  |  |
| Inferior temporal gyrus | Gyrus temporalis inferior | Gyrus temporal inférieur |  |  | T3 |
| Fusiform gyrus | Gyrus fusiformis | Gyrus fusiforme | Lateral occipitotemporal gyrus | Gyrus occipitotemporalis lateralis (Pansch) | T4 |
| Parahippocampal gyrus | Gyrus parahippocampalis | Gyrus parahippocampal |  |  | T5 |

**References:**

Arnold, F. (1838-1843). *Tabulae anatomicae, quas ad naturam accurate descriptas in lucem editi.* Zürich: Höhr.

Bischoff, T.L.W. (1868). *Die Grosshirnwindungen des Menschen mit Berücksichtigung ihres Entwickelung bei dem Foetus und ihrer Anordnung bei den Affen.* München.

Broca, P.P. (1878a). Nomenclature cérébrale: Dénomination et subdivision des hémisphères et des anfractuosités sur la surface. *Rev*. *Anthropol*. 2, 193-236.

Broca, P.P. (1878b). Anatomie comparée des circonvolutions cérébrales. Le grand lobe limbique et le scissure limbique dans le série des mammifères. *Rev*. *Anthropol*. 2, 385-498.

Burdach, K.F. (1822). *Vom* *Baue* *und* *Leben* *des* *Gehirns*, Bd 2. Leipzig: Dyk'schen Buchhandlung.

Charcot, J.M. (1876-1880). *Leçons sur les localisations dans les maladies du cerveau faites à la Faculté de Médecine de Paris* (1875), Paris.

Cruveilhier, J. (1844). *Anatomie* *descriptive*, Vol 4. Paris: Béchet Jeune.

Dejerine, J.J. (1895). *Anatomie* *des* *centres* *nerveux*, Vol 1. Paris: Rueff.

Duval, M. (1881-1882). Le corne d'Ammon. *Arch*. *Neurol*., Vols 2, 3, Paris.

Duvernoy, H.M. (1992). *Le* *cerveau* *humain*. Paris: Springer.

Eberstaller, O. (1884). Zur Oberflächenanatomie der Grosshirnhemisphären. *Wien*. *Med*. *Bl*. 7, 479-482, 542-582, 644-646.

Ecker, A. (1869). *Die* *Hirnwindungen* *des* *Menschen*. Braunschweig: Vieweg.

Foville, M. (1844). *Traité complet de l'anatomie, de la physiologie et de la pathologie du système nerveux cérébro-spinal.* Paris: Fortin, Masson et Cie..

Gerdy, P.N. (1838). Récherches sur l'encéphale. *J*. *Connaiss*. *Méd*.-*Chir*., 258-263 (Quoted from Swanson, 2014).

Gratiolet, L.P. (1854). *Mémoire sur les plis cérébraux ou l'homme et des primates.* Paris: Bertrand.

Henle, J. (1871). *Handbuch* *der* *Nervenlehre* *des* *Menschen*. Braunschweig: Vieweg.

Heschl, R.L. (1878). *Úber die vordere quere Schläfenwindung des menschlichen Gehirns.* Vienna: Braumüller.

Huschke, E. (1854). *Schädel, Hirn und Seele des Menschen und der Thiere nach Alter, Geschlecht und Race, dargestllt nach neuen Methoden und Untersuchungen.* Jena: Mauke.

Huxley, A. (1871). *A Manual of the Anatomy of Vertebrated Animals.* London.

Kéraval, P. (1884a). La synonymie des circonvolutions cérébrales de l'homme. I. Face externe et inférieure. *Arch*. *Neurol*., Tome VIII, 181-200.

Kéraval, P. (1884b). Ibid. II. Face interne. *Arch*. *Neurol*., Tome VIII, 314-320.

Meynert, T.H. (1867/68). Der Bau der Grosshirnrinde und seine örtlichen Verschiedenheiten, nebst einem pathologisch-anatomischen Corollarium. *Vierteljahrschr*. *Psychiat*. 1, 77-93, 125-217, 381-403; 2, 88-113.

Pansch, A.G. (1868). Úber die typische Anordnung der Furchen und Windungen auf den Grosshirnhemisphären des Menschen und der Affen. *Arch*. *Anthropol*. 3, 227-257.

Pansch, A.G. (1879). *Die Furchen und Wülste am Grosshirn des Menschen.* Berlin: Oppenheim.

Pozzi, S.J. (1873). Circonvolutions cérébrales. *Diction* *encyclopédie*.

Reil, J.C. (1809). Untersuchungen über den Bau des grossen Gehirns im Menschen. *Arch*. *Physiol*. 9, 136-208.

Schwalbe, G. (1881). *Lehrbuch* *der* *Neurologie*. Erlangen: Besold.

Swanson, L.W. (2014). *Neuroanatomical Terminology. A lexicon of classical origins and historical foundations.* New York: Oxford University Press.

Tarin, P. (1750). *Adversaria Anatomica prima. De omnibus cerebri, nervorum et organorum functionibus animalibus inserventium, descriptionibus et iconismis.* Paris.

Testut, L,, and Latarjet, A. (1948). *Traité* *d'anatomie* *humaine*, Vol 2. 9th ed. Paris: Doin.

Turner, W. (1891). The convolutions of the brain. A study in comparative anatomy. *J*. *Anat*. *Physiol*. 25, 105-153.

Valentin, G. (1841). *Hirn und Nervenlehre. Soemmerring's Vom Baue des menschlichen Körpers,* 4. Band. Leipzig: Voss.

Vicq d'Azyr, F. (1786). *Traité d'anatomie et de physiologie, avec des planches coloriées réprésentant au naturel les divers organes de l'homme et des animaux,* Tome I. Paris: Didot.
