# Supplementary material for: Toward a Common Terminology for the Gyri and Sulci of the Human Cerebral Cortex
Source: Front Neuroanat. 2018 Nov 19;12:93. doi: 10.3389/fnana.2018.00093 (PMC6252390; doi:10.3389/fnana.2018.00093)
Supplement: Supplementary file 2 [file Table_2.DOCX]

**Supplementary Table 2: Synonyms and eponyms for the main cerebral sulci (based on data by Kéraval, 1884a, b; Dejerine, 1895; Testut and Latarjet,1948; and Swanson, 2014)**

| **English official term** | **TNA Latin term** | **French term (from Duvernoy 1992)** | **English, French and German synonyms with sources** | **Latin synonyms with sources** | **Eponyms** |
| --- | --- | --- | --- | --- | --- |
| **Sulci on lateral surface** |  |  |  |  |  |
| **Interlobar sulci** |  |  |  |  |  |
| Lateral sulcus | Sulcus lateralis | Scissure latérale | Grande scissure interlobaire (Chaussier); Scissure de Sylvius (Broca, Charcot); Fissure of Sylvius (Turner, Ferrier); Lateral fissure (Huxley); Lateral- oder Sylvische Spalte (Pansch, Schwalbe) | Fissura Sylvii (Eberstaller, Pansch); Fissura lateralis (Henle); Fissura sive fossa Sylvii (Ecker) | **Sylvius** |
| Central sulcus | Sulcus centralis | Scissure centrale | Scissure de Rolando (Leuret, Giacomini, Broca); Postero-parietal sulcus (Huxley);Fissure of Rolando (Turner); Centralfurche (Huschke, Ecker); Centralspalte (Pansch, Schwalbe) | Sulcus centralis (Ecker), Fissura centralis (Eberstaller); Fissura transversa anterior (Pansch); | **Rolando** |
| Parietooccipital sulcus | Sulcus parietooccipitalis | Scissure pariéto-occipitale | Scissure perpendiculaire externe (Gratiolet); Scissure occipitale (Broca); Occipito-parietal fissure (Turner); Affenspalte (Schwalbe) | Fissura parietooccipitalis (Eberstaller); Pars superior sive lateralis fissurae parietooccipitalis (Ecker); Fissura occipitalis perpendicularis (Henle, Bischoff); Fissura occipitalis (Pansch) |  |
| Preoccipital notch | Incisura preoccipitalis |  |  |  | **Meynert** |
| **Lobar sulci** |  |  |  |  |  |
| Superior frontal sulcus | Sulcus frontalis superior | Sillon frontal supérieur | Premier sillon frontal (Broca, Dejerine); Scissure frontale supérieure (Pozzi); Superofrontal sulcus (Huxley, Turner);  Obere Stirnfurche (Ecker, Pansch) | Sulcus frontalis superior (Ecker, Pansch) |  |
| Inferior frontal sulcus | Sulcus frontalis inferior | Sillon frontal inférieur | Deuxième sillon frontal (Broca, Dejerine); Scissure frontale inférieure ou sourcilière (Pozzi); Inferofrontal sulcus (Huxley); Untere Stirnfurche (Ecker, Pansch) | Sulcus frontalis inferior (Ecker); Sulcus frontalis medius (Pansch) |  |
| Precentral sulcus | Sulcus precentralis | Sillon précentral inférieur, parallel to the Central sulcus, with a small Sillon précentral supérieur | Sillon prérolandique (Broca); Scissure parallèle frontale (Pozzi); Anteroparietal sulcus (Huxley); Ascending limb of Sylvian fissure (Turner); Precentralfurche (Ecker, Pansch) | Sulcus precentralis (Ecker); Sulcus frontalis medius, Ramus descendens (Pansch) |  |
| Intraparietal sulcus | Sulcus intraparietalis | Sillon intraparietal; Segment ascendant, Segment horizontal, and Segment descendant | Sillon pariétal (Broca); Intraparietal fissure (Turner) | Sulcus retrocentralis (Eberstaller); Sulcus interparietalis (Ecker); Sulcus occipitoparietalis (Schwalbe); Sulcus parietalis (Pansch) | **Turner** |
| Superior temporal sulcus | Sulcus temporalis superior | Sillon temporal supérieur | Scissure parallèle (Gratiolet); Premier sillon temporal (Broca, Dejerine); First temporal sulcus, Anterotemporal sulcus (Huxley); Parallel fissure (Turner); Erste Schläfenfurche (Bischoff, Pansch) | Sulcus temporalis superior (Ecker, Pansch) |  |
| Inferior temporal sulcus | Sulcus temporalis inferior | Sillon temporal inférieur | Deuxième sillon temporal (Broca, Dejerine); Second temporal sulcus; Middle temporal sulcus, Posterotemporal sulcus (Huxley); Zweite Schläfenfurche (Bischoff, Pansch) | Sulcus temporalis medius (Ecker) |  |
|  |  |  |  |  |  |
| **Sulci of inferomedial surface** |  |  |  |  |  |
| **Interlobar sulci** |  |  |  |  |  |
| Cingulate sulcus | Sulcus cinguli | Sillon cingulaire | Scissure sous-frontale (Broca); Scissure festonneé (Pozzi); Grand sillon du lobe fronto-pariétal (Gratiolet); Sillon du corps calleux (Gromier); Calloso-marginal sulcus (Turner, Huxley) | Fissura callosomarginalis (Eberstaller); Sulcus calloso-marginalis (Bischoff); Sulcus medialis fronto-parietalis (Pansch) |  |
| Parietooccipital sulcus | Sulcus parietooccipitalis | Scissure pariéto-occipitale | Scissure perpendiculaire interne (Gratiolet); Occipitoparietal fissure (Huxley); Scissure occipitale interne (Broca) | Fissura posterior (Burdach, Arnold); Fissura occipitalis internus (Pansch); Fissura occipitalis perpendicularis interna (Bischoff; Ecker) | **Gratiolet** |
| Collateral sulcus | Sulcus collateralis | Sillon collatéral; Sillon occipitotemporal médial | Sillon occipitotemporal médial; Sillon temporo-occipital interne (Gratiolet); Quatrième sillon temporal (Broca); Deuxième scissure temporo-occipital (Pozzi); Medial occipitotemporal sulcus (Turner); Fourth temporal sulcus; Collateral fissure (Huxley, Turner); Inferior longitudinalis sulcus (Huxley) | Fissura collateralis sive temporalis inferior (Bischoff); Sulcus longitudinalis inferior (Huschke); Sulcus occipitotemporalis inferior (Ecker) |  |
| Rhinal sulcus | Sulcus rhinalis | Sulcus rhinalis |  | Sulcus rhinicus (Retzius) |  |
| Hippocampal sulcus | Sulcus hippocampalis | Sillon hippocampal | Rainure du grand hippocampe (Broca); Dentate fissure (Turner) | Fissura hippocampi (Ecker) |  |
| **Lobar sulci** |  |  |  |  |  |
| Calcarine sulcus | Sulcus calcarinus | Sillon calcarin | Scissure des hippocampes (Gromier); Partie postérieure de la scissure des hippocampes (Gratiolet); Scissure calcarine (Broca); Calcarine fissure (Turner); Kleinere hintere Grube (Reil); Furche unter dem Zwickel (Burdach) | Fissura calcarina (Eberstaller, Ecker); Fissura hippocampi (Bischoff); Fissura horizontalis (Pansch); Fissura occipitalis horizontalis (Henle) |  |
| Olfactory sulcus | Sulcus olfactorius | Sillon olfactif; Sillon orbitaire médial | Sillon du nerve olfactiv (Vicq d'Azyr); Scissure olfactive (Giacomini); Sillon droit ou premier sillon orbitaire (Broca); Olfactory sulcus (Quain, Turner) | Sulcus rectus (Meynert); Sulcus olfactorius (Ecker, Bischoff, Henle, Pansch) |  |
| Orbital sulci | Sulci orbitales | Sillon orbitaire en H;  Sillon orbitaire en arqué | Solco crociforme (Rolando); Scissure orbitaire (Giacomini); Deuxième sillon orbitaire (Broca); Triradiate sulcus (Turner) | Sulcus orbitalis (Ecker); Sulcus cruciatus (Meynert) |  |
| Occipitotemporal sulcus | Sulcus occipitotemporalis | Sillon occipitotemporal latéral | Lateral occipitotemporal sulcus; Inferior temporal sulcus; Third temporal sulcus; Sillon temporo-occipital externe (Gratiolet); Troisième sillon temporal (Broca) | Sulcus temporo-occipitalis (Ecker) |  |

**References:**

Arnold, F. (1838-1843). *Tabulae anatomicae, quas ad naturam accurate descriptas in lucem editi.* Zürich: Höhr.

Bischoff, T.L.W. (1868). *Die Grosshirnwindungen des Menschen mit Berücksichtigung ihres Entwickelung bei dem Foetus und ihrer Anordnung bei den Affen.* München.

Broca, P.P. (1878a). Nomenclature cérébrale: Dénomination et subdivision des hémisphères et des anfractuosités sur la surface. *Rev*. *Anthropol*. 2, 193-236.

Broca, P.P. (1878b). Anatomie comparée des circonvolutions cérébrales. Le grand lobe limbique et le scissure limbique dans le série des mammifères. *Rev*. *Anthropol*. 2, 385-498.

Burdach, K.F. (1822). *Vom Baue und Leben des Gehirns,* Bd 2. Leipzig: Dyk'schen Buchhandlung.

Charcot, J.M. (1876-1880). *Leçons sur les localisations dans les maladies du cerveau faites à la Faculté de Médecine de Paris* (1875), Paris.

Chaussier, F. (1807). *Exposition sommaire de la structure et des differentes parties de l'encéphale ou cerveau.* Paris: Barrois.

Dejerine, J.J. (1895). *Anatomie* *des* *centres* *nerveux*, Vol 1. Paris: Rueff.

Duvernoy, H.M. (1992). *Le* *cerveau* *humain*. Paris: Springer.

Eberstaller, O. (1884). Zur Oberflächenanatomie der Grosshirnhemisphären. *Wien*. *Med*. *Bl*. 7, 479-482, 542-582, 644-646.

Ecker, A. (1869). *Die* *Hirnwindungen* *des* *Menschen*. Braunschweig: Vieweg.

Ferrier, D. (1876). The localization of functions in the brain. *Proc*. *Roy*. *Soc*. 22, 229-232.

Giacomini, C.H. (1883). Fascia dentata del grand ippocampo nel cervello umano. *G*. *Accad*. *Med*. *Torino*, Vol 31.

Gratiolet, L.P. (1854). *Mémoire sur les plis cérébraux ou l'homme et des primates.* Paris: Bertrand.

Gromier, J. (1874). *Étude sur les circonvolutions cérébrales chez l'homme et chez les singes.* Thèse pour le doctorat en médecin. Paris: Parent.

Henle, J. (1871). *Handbuch* *der* *Nervenlehre* *des* *Menschen*. Braunschweig: Vieweg.

Huschke, E. (1854). *Schädel, Hirn und Seele des Menschen und der Thiere nach Alter, Geschlecht und Race, dargestllt nach neuen Methoden und Untersuchungen.* Jena: Mauke.

Huxley, A. (1871). *A Manual of the Anatomy of Vertebrated Animals.* London.

Kéraval, P. (1884a). La synonymie des circonvolutions cérébrales de l'homme. I. Face externe et inférieure. *Arch*. *Neurol*., Tome VIII, 181-200.

Kéraval, P. (1884b). Ibid. II. Face interne. *Arch*. *Neurol*., Tome VIII:314-320.

Leuret, F., and Gratiolet, L.P. (1839-1857). *Anatomie comparée du système nerveux consideré dans ses rapports avec l'intelligence.* 2 Vols and Atlas, Paris: Baillière et Fils.

Meynert, T.H. (1867/68). Der Bau der Grosshirnrinde und seine örtlichen Verschiedenheiten, nebst einem pathologisch-anatomischen Corollarium. *Vierteljahrschr*. *Psychiat*. 1, 77-93, 125-217, 381-403; 2, 88-113.

Pansch, A.G. (1868). Úber die typische Anordnung der Furchen und Windungen auf den Grosshirnhemisphären des Menschen und der Affen. *Arch*. *Anthropol*. 3, 227-257.

Pansch, A.G. (1879). *Die Furchen und Wülste am Grosshirn des Menschen.* Berlin: Oppenheim.

Pozzi, S.J. (1873). Circonvolutions cérébrales. *Diction* *encyclopédie*.

Quain, J. (1834). *The* *Elements* *of* *Anatomy*, 3rd ed. London: Taylor.

Reil, J.C. (1809). Untersuchungen über den Bau des grossen Gehirns im Menschen. *Arch*. *Physiol*. 9, 136-208.

Retzius, G. (1896). *Das Menschenhirn: Studien in der makroskopischen Morphologie.* Stockholm: Norstedt.

Rolando, L. (1809). *Saggio sopra la vere struttura del cervello dell'uomo e degl'animali e sopra le funzioni del sistema nervoso.* Sassari.

Rolando, L. (1829). Della struttura degli emisferi cerebrali. *Mem*. *Reg*. *Accad*. *Sci*. *Torino*.

Schwalbe, G. (1881). *Lehrbuch* *der* *Neurologie*. Erlangen: Besold.

Swanson, L.W. (2014). *Neuroanatomical Terminology. A lexicon of classical origins and historical foundations.* New York: Oxford University Press.

Testut, L., and Latarjet, A. (1948). *Traité* *d'anatomie* *humaine*, Vol 2. 9th ed. Paris: Doin.

Turner, W. (1891). The convolutions of the brain. A study in comparative anatomy. *J*. *Anat*. *Physiol*. 25, 105-153.

Vicq d'Azyr, F. (1786). *Traité d'anatomie et de physiologie, avec des planches coloriées représentant au naturel les divers organes de l'homme et des animaux,* Tome I. Paris: Didot.
